# Porcine familial adenomatous polyposis model enables systematic analysis of early events in adenoma progression

**DOI:** 10.1038/s41598-017-06741-8

**Published:** 2017-07-26

**Authors:** Tatiana Flisikowska, Monika Stachowiak, Hongen Xu, Alexandra Wagner, Alejandra Hernandez-Caceres, Christine Wurmser, Carolin Perleberg, Hubert Pausch, Anna Perkowska, Konrad Fischer, Dmitrij Frishman, Ruedi Fries, Marek Switonski, Alexander Kind, Dieter Saur, Angelika Schnieke, Krzysztof Flisikowski

**Affiliations:** 1 Chair of Livestock Biotechnology, Technische Universität München, Freising, Germany; 2 Department of Genetics and Animal Breeding, Poznan University of Life Sciences, Poznan, Poland; 3 Department of Bioinformatics, Wissenschaftszentrum Weihenstephan, Technische Universität München, Freising, Germany; 4 Chair of Animal Breeding, Technische Universität München, Freising, Germany; 5 Institute of Bioinformatics and Systems Biology, Helmholtz Zentrum Munich - German Research Center for Environmental Health, Neuherberg, Germany; 6 St Petersburg State Polytechnic University, St Petersburg, Russia; 7 Klinikum Rechts der Isar II, Technische Universität München, Munich, Germany

## Abstract

We compared gene expression in low and high-grade intraepithelial dysplastic polyps from pigs carrying an *APC* ^*1311*^ truncating mutation orthologous to human *APC* ^*1309*^, analysing whole samples and microdissected dysplastic epithelium. Gene set enrichment analysis revealed differential expression of gene sets similar to human normal mucosa versus T1 stage polyps. Transcriptome analysis of whole samples revealed many differentially-expressed genes reflecting immune infiltration. Analysis of microdissected dysplastic epithelium was markedly different and showed increased expression in high-grade intraepithelial neoplasia of several genes known to be involved in human CRC; and revealed possible new roles for *GBP6* and *PLXND1*. The pig model thus facilitates analysis of CRC pathogenesis.

## Introduction

Functional disruption of the adenomatous polyposis coli (*APC*) tumour suppressor initiates formation of most adenomas in the human gut, and is responsible for most cases of familial adenomatous polyposis (FAP), an inherited predisposition to colorectal cancer (CRC)^1^. While the severity of the condition varies considerably, FAP patients typically develop tens to hundreds of adenomatous polyps in the colon and rectum in early life and have greatly increased risk of developing CRC^2^.

Several genetic and epigenetic alterations have been implicated in this progression, including tumour suppressor genes and proto-oncogenes (*TP53, KRAS*, *BRAF, SMAD2/4, PIK3CA*), chromosomal instability, aberrant DNA methylation and histone modification^3^. However the events that determine whether an early stage polyp proceeds towards cancer have not yet been identified. As Sievers *et al*.^4^ recently reported, human precancerous polyps are very heterogeneous, which hinders comparative molecular analyses.

We investigated gene expression in the premalignant progression of dysplastic polyps in pigs carrying an engineered translational stop signal at codon 1311 in the *APC* gene (*APC* ^*1311*^) orthologous to a human *APC* ^*1309*^ mutation responsible for FAP^5^, to determine if molecular changes in the pig model parallel events in small polyps in humans, and whether pigs can be used to reveal new events in early CRC development. The value of pigs in modeling CRC and other cancers has been discussed in several publications e.g. refs 6, 7. The *APC* ^*1311*^ pig model has the advantage that samples can be taken from many individuals with the same initiating mutation, avoiding an important source of variation. Changes held in common across such samples are more likely to have a function in premalignant transformation.

## Results and Discussion

### Phenotypic characterisation of APC^1311/+^ pigs

**Figure 1:**
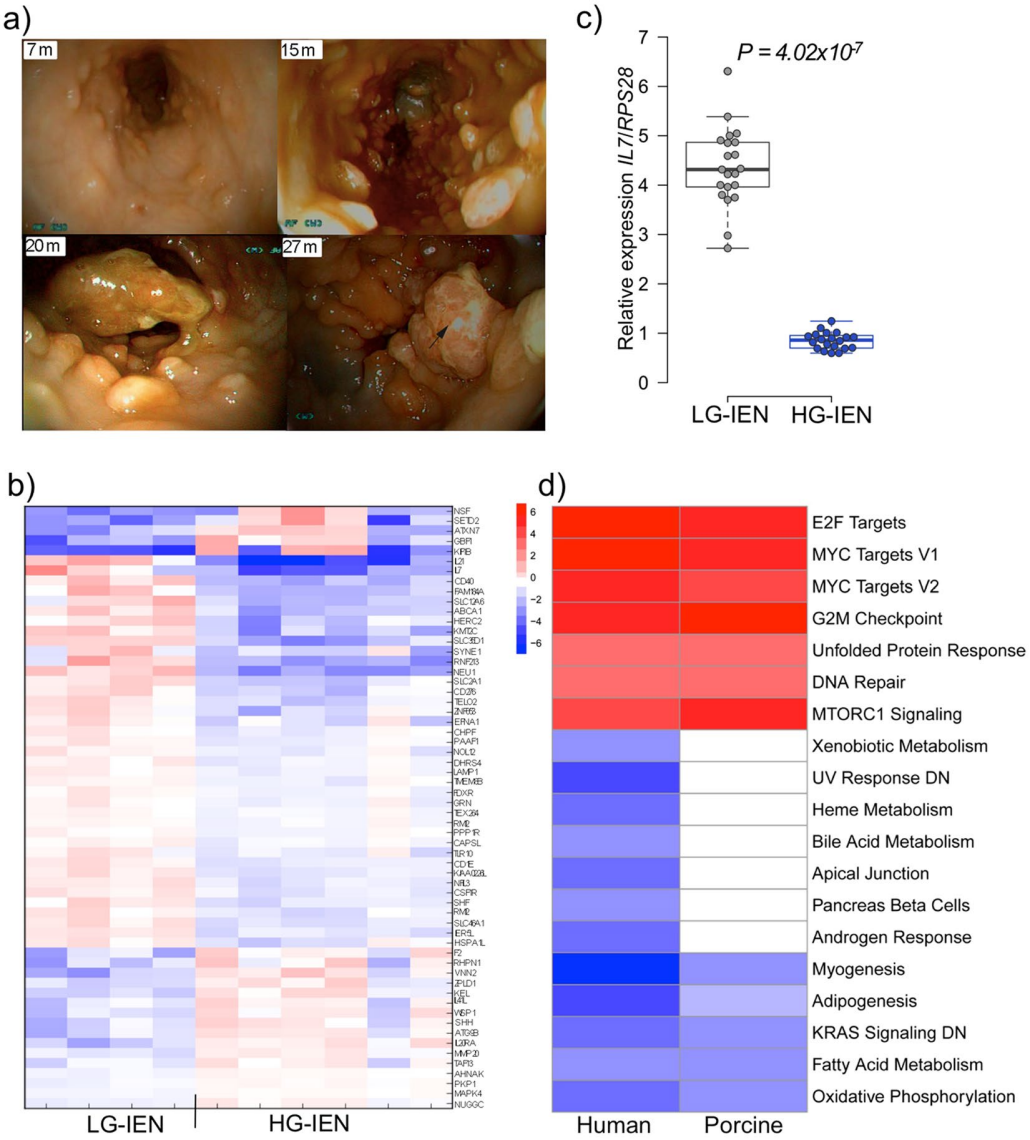
Polyposis and transcriptome patterns specific for low- (LG-IEN) and high-grade intraepithelial (HG-IEN) dysplasia in polyps of *APC* ^*1311/*+^ pigs. (**a**) Endoscopic images of an F1 *APC* ^*1311/*+^ animal (ID 73). Photographs were taken at approximately the same location (~15 cm depth) at ages 7, 15, 20 and 27 months as indicated. The tumour shown at 20 and 27 months was ~2.5 cm diameter, and at the later date had become hardened and more irregular. (**b**) Heatmap of the top 60 genes showing transcriptome pattern specific for LG-IEN and HG-IEN whole samples ranked by normalised expression differences between the two groups and P-value. (**c**) Illustrative quantitative PCR analysis of *IL7* in LG-IEN and HG-IEN polyps (n = 20 per group, P = 4.02 × 10^−7^). qPCR measurements were normalised to porcine normal colonic mucosa samples (n = 6). (**d**) Gene set enrichment analysis for human microsatellite stable (MSS) T1 stage polyps versus human normal colonic mucosa from TCGA database and LG-IEN versus HG-IEN colon polyps in *APC* ^*1311/*+^ pigs.

To enable systematic investigation of CRC and its precursor lesions, we previously generated pigs carrying an *APC* ^*1311*^ mutation, orthologous to human *APC* ^*1309*^ responsible for a severe form of FAP^5^. Four generations of *APC* ^*1311/*^^+^pigs have now been examined regularly by colonoscopy (Fig. 1a). Findings to date have confirmed that the *APC* ^*1311*^ FAP model recapitulates key aspects of the human disease, most notably multiple polyps in the colon and rectum^5^. Polyps have not so far been observed in other parts of the pig gastrointestinal tract. We also observe that, as in human patients^8^, polyp severity varies between individual pigs carrying the same germ line mutation. Histological evaluation of biopsy samples reveals typical epithelial features of the adenoma-carcinoma sequence including aberrant crypt foci, adenomatous polyps with low (LG-IEN) and high-grade intraepithelial (HG-IEN) dysplasia and carcinoma *in situ* (Figure S1).

### Comparison of LG-IEN and HG-IEN polyps by gene expression analysis of whole biopsy samples

The progress of polyposis in *APC* ^*1311/*+^ pigs shows clear morphological, histological and immunohistological similarities to human CRC^5^. To gain insight into the transition from LG-IEN and HG-IEN, we investigated transcriptional changes occurring in early polyps in the pig model. HG-IEN is the more advanced stage, where the risk of malignancy increases considerably^9^. Whole biopsy samples from 10 polyps, each ~1 cm diameter, taken from six individual pigs at sites from 20 to 40 cm deep, histologically classified as either LG-IEN (n = 4) or HG-IEN (n = 6), were analysed by next generation RNA sequencing, and transcriptome clusters specific for LG-IEN and HG-IEN polyps identified. More than 300 genes were expressed at significantly different levels between the two groups (*P* < 0.01, Fig. 1b, Table S1), most of which (n = 192) showed lower expression in HG-IEN than LG-IEN polyps. Reported functions of genes in this subset include metabolic processes (*MMP20*, *TELO2*, *HERC2, MAPK4, S100A8, S100A9*), intracellular transport (*TMEM8B*, *SLC12A6, SLC46A1*), and many were immune related (*IL7*, *IL17D*, *CD276*, *IL21*, *IL20RA*, *CD40*). Differential expression of selected genes was validated by quantitative PCR (qPCR) (e.g. Fig. 1c, Table S2). The gene *AHNAK* showed the greatest reduction in HG-IEN polyps (3.3 fold; *P* = 4 × 10^−8^). *AHNAK* has been reported as a negative regulator of cell growth, and as a tumour suppressor that potentiates TGFβ signalling^10^. Inactivation of epithelial TGFβ signalling promotes development of intestinal cancer in mouse and human colon cells *ex vivo* ^11, 12^. Of those genes expressed at higher level in HG-IEN polyps, the stress-responsive gene *HSPA1L* (*P* = 1.7 × 10^−6^), a member of the HSP70 family, showed the greatest difference (3.6 fold). Others had functions related to Wnt signalling (*WISP1*), the TNF pathway (*SLC12A6*), and cell apoptosis and tumour microenvironment (*S100A8, S100A9*). Pathway analysis of differentially expressed genes using PANTHER software revealed that the histamine H1 receptor mediated signalling and B cell activation pathways were significantly over-represented (P < 0.05, Bonferroni corrected for multiple testing). Histamine receptors are known to play an important role in cancer development including CRC^13^. It should however be pointed out that most genes studied (n = 238) were unclassified in the pathways, which is a limitation when analysing the pig genome.

To assess the relevance of our animal model to human disease, we carried out gene set enrichment analysis (GSEA)^14^. Equivalent data from human LG-IEN versus HG-IEN polyps was not available, precluding direct side-by-side comparison with the pig. Differential expression data for human T1 stage microsatellite stable (MSS) and microsatellite instable (MSI) polyps versus normal colonic mucosa from The Cancer Genome Atlas (TCGA) database provided the closest comparison. GSEA analysis of pig data revealed that 25 of 50 gene sets showed significant differences (q value < 0.05) between LG-IEN and HG-IEN, including many sets important for tumorigenesis. Parallel analysis of the human MSS data showed 26 of 50 gene sets with q < 0.05, of which 19 were held in common with pig; human MSI data showed 23 of 50 gene sets with q < 0.05, of which 7 were held in common with pig, GSEA results are summarised in Fig. 1d. It is known that *APC* mutations are frequent in non-hypermutated (MSS) colon cancers^15^. There was a striking degree of similarity between changes in gene sets in pig and human MSS polyps and several close parallels in key functional sets, e.g. upregulation of MYC target genes, the E2F group of genes involved in cell cycle regulation and DNA synthesis, and the cell cycle G2/M checkpoint DNA damage gene set, and downregulation of interferon alpha and gamma response targets. This was clearly only a broad comparison and there were some anomalies, such as the apparent lack of Wnt pathway upregulation T1 human polyps despite its known role in polyposis. This latter is probably explained by the heterogeneous nature of the human data and the likely high proportion of stroma in the human polyp biopsies masking the contribution of tumour cells^16^.

These findings strongly suggest that similar molecular changes are involved in early polyp development in the *APC* ^*1311*^ FAP pig and in humans.

### Assessing severity grading by gene expression profile

It has been suggested that global gene expression profiles might be used to classify human CRC subtypes^17, 18^. To investigate whether transcriptome data is useful for assessing severity of polyposis, we performed RNA sequencing on a series of histologically unclassified 1 cm diameter polyps taken from 22 *APC* ^*1311/*+^ animals. Polyp biopsies were taken from animals with very low (<10 in distal 40 cm of colon and rectum) and very high (>100 in same region) numbers of colorectal polyps. Unsupervised clustering of RNA sequencing data revealed two distinct gene expression profiles (Figure S2). Again, of the 50 genes showing the greatest difference in expression between the two profiles, 18 were cytokines. The two gene expression clusters closely resembled those observed for histologically classified LG-IEN and HG-IEN polyps and, interestingly, correlated with severity of polyposis in the source animals. Nearly all samples (11 of 12) showing the HG-IEN profile were collected from *APC* ^*1311/*+^ pigs with a high number of polyps. A similar correlation between polyposis severity and the proportion of high grade dysplasia has also been reported in human FAP patients^9^.

### Immune cells make a significant contribution to transcriptome data using whole biopsies

As mentioned, a substantial proportion (18%) of genes with the most marked differences in expression between pig LG-IEN and HG-IEN were immune-related or known to be specific to immune cells. Immunohistochemical analysis of polyp samples confirmed infiltration of CD3+, CD4+, and CD8+ T cells (Figure S4). The role of immune cells in CRC is well documented, several studies have reported that cytokines produced by immune cells influence CRC progression^19^ and are linked to poor prognosis at early stages^20^. Our findings indicate a similarly important role in the pig model. However the presence of infiltrating immune cells in whole samples is likely to obscure transcriptome changes in the dysplastic epithelium.

### Comparison of microdissected LG-IEN and HG-IEN adenoma by gene expression analysis

**Figure 2:**
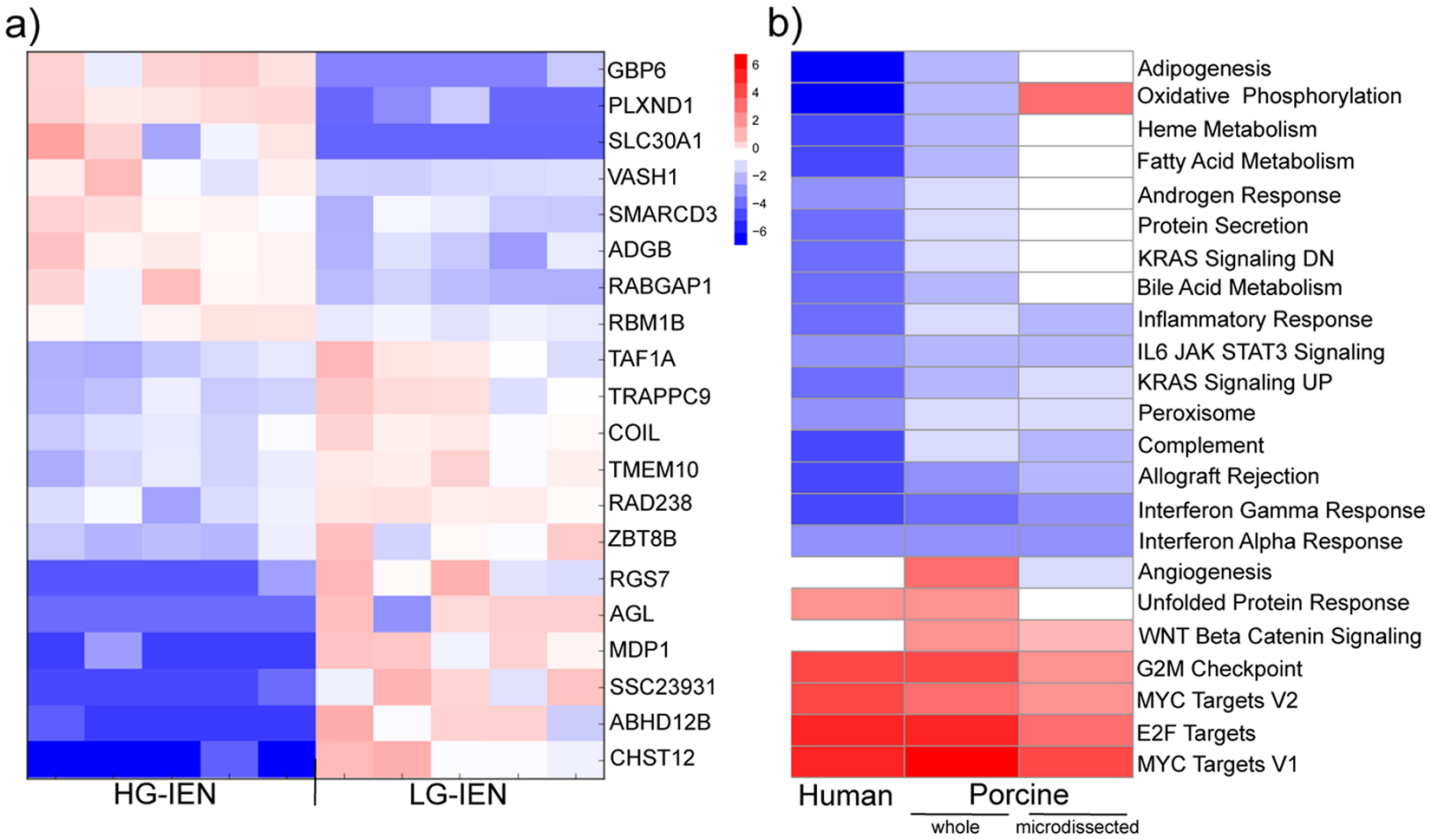
Transcriptome pattern specific for microdissected low- (LG-IEN) and high-grade intraepithelial (HG-IEN) adenomas in polyps of *APC* ^*1311/*+^ pigs. (**a**) Heatmap of top 20 genes showing transcriptome pattern specific for microdissected LG-IEN and HG-IEN. (**b**) Comparison of gene set enrichment analysis for human T1 stage polyps versus normal colonic mucosa; porcine LG-IEN versus HG-IEN polyp whole samples; and microdissected porcine LG-IEN and HG-IEN.

To restrict our analysis to events in the epithelium, we microdissected dysplastic epithelial cells from five distinct and separate LG-IEN and similarly five HG-IEN adenomas, each from the same polyps, and again performed RNA sequencing (Fig. 2a, Table S3). To our knowledge, similar studies have not been performed on human adenomas, although Lechner *et al*.^21^ examined a limited number of cancer-related genes in microdissected human LG-IEN polyps. Our analysis of microdissected samples revealed marked differences to results from whole samples. Several genes identified as differentially expressed in the whole samples were not represented, e.g. *IL7*, *IL17D*, *S100A8, S100A9, AHNAK*, indicating that such transcriptional changes occurred in other tissues, such as stroma and infiltrating immune cells. New gene expression differences were also revealed by the more defined analysis. Indeed, none of the top 20 genes identified as differentially expressed in microdissected epithelium were significant in the whole sample analysis. Most notable were higher expression of *PLXND1* and *GBP6* in HG-IEN (>2- fold, *P* < 3 × 10^−6^; Table S4). While the top gene *PLXND1* did show as differentially expressed in whole biopsy samples, this fell below statistical significance after correction for multiple testing. Analysis of gene expression data from both Gene Expression Omnibus and TCGA databases revealed that *PLXND1* expression decreases in later stages of CRC^22, 23^, but *GBP6* showed no significant expression changes. *PLXND1* encodes a transmembrane signal transduction receptor known to bind semaphorins and other ligands. It is upregulated in a wide variety of human tumour cells^22^ and has been reported as mediating epithelial-to-mesenchymal transition in ovarian cancer^24^, and associated with tumour invasiveness and metastasis in human CRC^25^. *GBP6* belongs to the family of guanylate binding proteins and is best known as a gene induced by interferon-γ signalling. Interferon-γ is produced predominantly by NK and T-cells, so increased *GBP6* expression in HG-IEN may reflect interplay between the epithelium and infiltrating immune cells. *GBP6* has not previously been implicated in tumorigenesis although a related gene, GBP1, is involved in Interferon-γ dependent CRC progression^26^. Other genes expressed at higher level in HG-IEN have reported functions in oxidative stress and anti-inflammatory activity *(SLC30A1)*, p53 regulation and cell proliferation *(VASH1)*, Wnt pathway regulation *(SMARCD3)*, and DNA binding *(RABGAP1*, *RBM18)*. Of those genes expressed at lower level in HG-IEN, the greatest differences (>1.75- fold, *P* < 2.08 × 10^−6^) were in *RAD23B*, a DNA repair enzyme, and *CHST12* (Table S4). *CHST12* encodes a carbohydrate sulphotransferase involved in extracellular matrix formation and its reduction in later stage dysplasia may reflect disruption of extracellular matrices as tissue organisation is affected. Reduced expression of other members of this family has been associated with invasiveness in liver carcinoma cells^27^.

GSEA of microdissected epithelium revealed a more restricted set of changes than in whole samples. Of those with q value < 0.05, five gene sets were upregulated in HG-IEN, all of which were also highlighted in whole biopsy samples, and eight gene sets showed downregulation in HG-IEN. Eight gene sets identified in whole samples showed no change in the microdissected epithelium, e.g. unfolded protein response, bile acid metabolism, adipogenesis, fatty acid and haeme metabolism (Fig. 2b), suggesting that such transcriptome changes occur in other components. The oxidative phosphorylation gene set was more highly expressed in microdissected than in whole HG-IEN samples. Again this can probably be explained by the exclusion of immune cells. Immune cells such as T cells are known to downregulate oxidative phosphorylation in favour of glycolysis^28^.

### Analysis of allele specific expression

**Figure 3:**
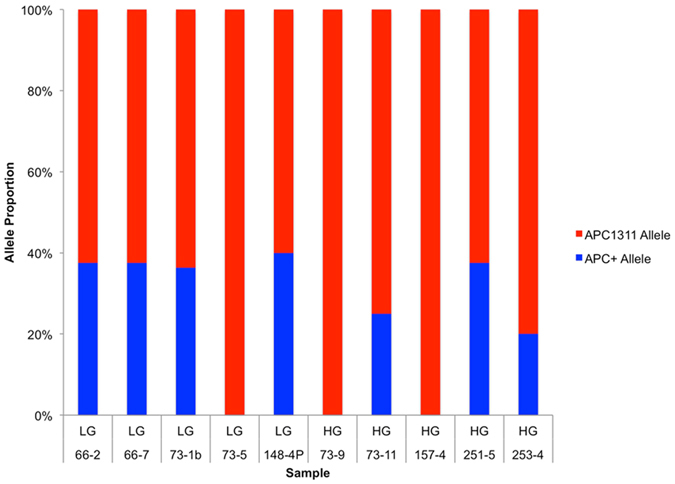
Proportion of *APC* ^*1311*^ and *APC* ^+^ allele expression in LG-IEN and HG-IEN whole samples. Mutant and wild type *APC* alleles can be distinguished by the replacement of G by T in codon 1311 (GAA), which introduces a premature stop codon. The allele proportion was based on raw RNA sequencing reads mapped in this region.

**Figure 4:**
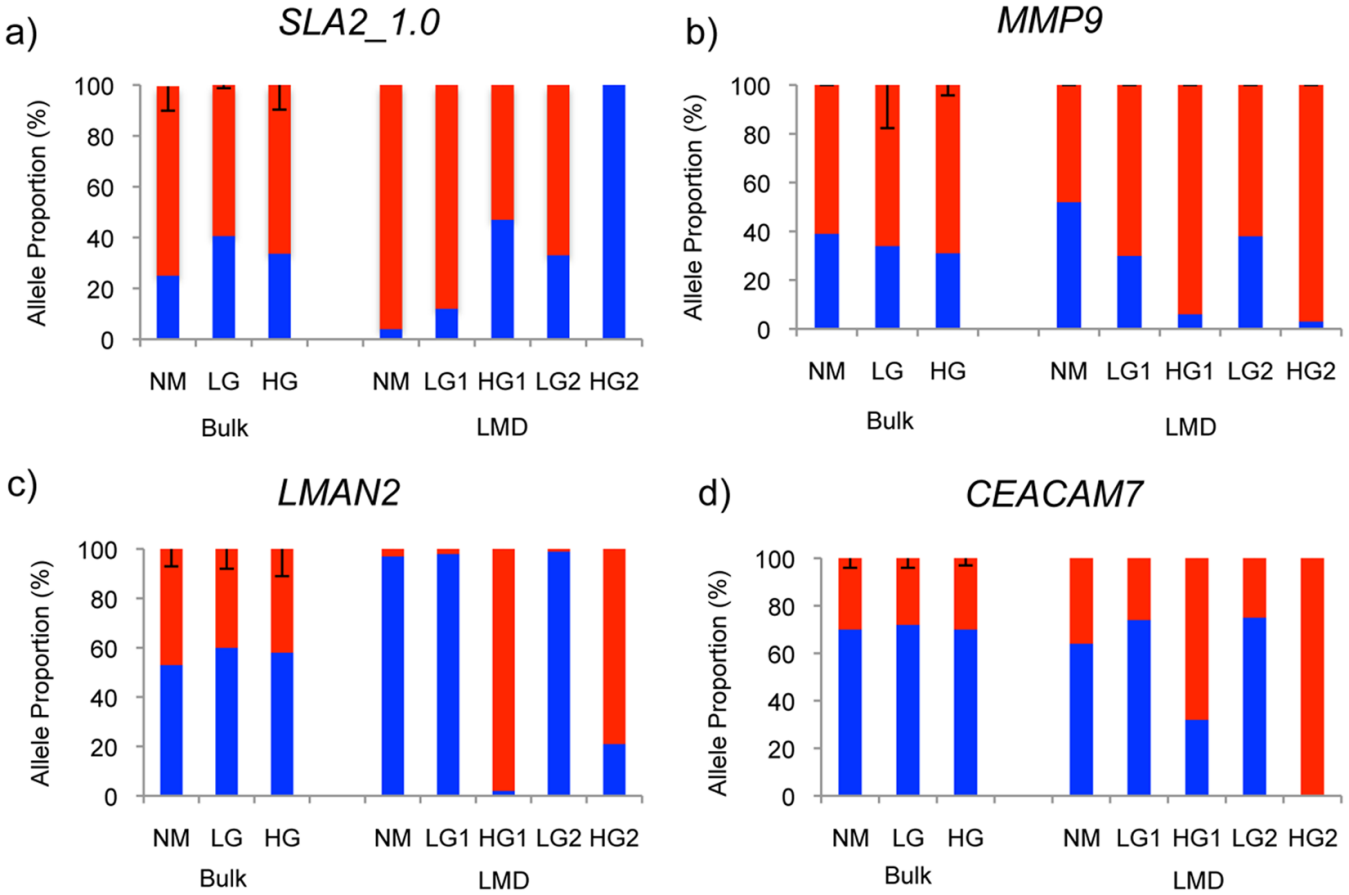
Allele specific expression of selected genes in whole and microdissected samples quantified using pyrosequencing. LG1, LG2, HG1, HG2 represent microdissected adenomas from the same polyp. The following SNPs were analysed: *MMP9* - rs341963098A/G; *SLA2_1.0* rs337323048A/G; *LMAN2* – rs339673049C/T; *CEACAM7* – 6:45194986 C/T. All SNP positions are according to the Sscrofa10.2 porcine genome reference sequence.

Changes in cell subpopulations over time are a common feature of many tumours, and can be monitored by measuring the proportional expression of different alleles of the same gene. Allele specific expression (ASE) is reportedly common in CRC^29^, but early polyps have not so far been investigated. Analysis of our RNA sequencing data from whole samples revealed that, as expected, the mutant *APC* ^*1311*^ allele was expressed at higher level than the wild-type *APC* allele^5, 30^ (Fig. 3). We then quantified transcriptome-wide ASE in the LG-IEN and HG-IEN polyp whole samples. Variant calling revealed 705,000 heterozygous SNPs, of which 48,000 (6%) showed allelic imbalance in at least one sample either in LG-IEN and HG-IEN polyps (*PvalueAdj* <0.05, Table S5). Imbalanced allele expression was detected for SNPs in several cancer-related genes such as *EpCAM*, *MSH2*, *MMP7*, *MMP12*, *PLOR1D* and *CCL5*. To specifically investigate the dysplastic epithelium, we selected genes with known function in epithelium and CRC progression (*MMP9, CEACAM7, LMAN2*) or immune cells (*SLA2*) and analysed allele expression in microdissected samples from distinct LG-IEN and HG-IEN adenoma from the same polyp (Fig. 4). The allele proportion of SNPs analysed in LG-IEN more closely resembled normal mucosa than did SNPs in HG-IEN. These differences were more evident in microdissected epithelium than in whole samples, consistent with the emergence of cell subpopulations in HG-IEN even at this premalignant stage. Reduced detection of one allele by PCR, as observed for SNPs in the *MMP9* and *LMAN2* genes in HG-IEN polyps, suggests overgrowth of cells carrying that allele by other faster growing cells with the other allele. These ASE results for HG-IEG polyps are consistent with the high frequency of chromosomal imbalances reported for MSS colorectal cancers^31^.

## Conclusions

In summary, pigs genetically predisposed to colorectal polyposis based on a single, well-defined genetic mutation enable systematic analysis of CRC precursor lesions, offering an important complement to the study of human patients and existing genetically-modified mouse models. Gene expression data from whole samples obtained by biopsy of polyps from *APC* ^*1311*^ pigs correlates with alterations in molecular pathways involved in early pathogenesis of human colorectal cancer. Microdissection and specific analysis of the epithelial component revealed that many transcriptome changes apparent in whole samples actually reflect events outside the epithelium, notably immune infiltration. Allele-specific expression was clearly evident, especially in microdissected HG-IEN, consistent with the emergence of cell subpopulations. Analysis of microdissected dysplastic epithelium also revealed possible roles for cancer-related genes not previously associated with CRC (*GBP6*, *PLXND1*) in early stage adenoma progression.

## Material and Methods

### Animals

Animal experiments were approved by the Government of Upper Bavaria (permit number 55.2-1-54-2532-6-13) and performed according to the German Animal Welfare Act and European Union Normative for Care and Use of Experimental Animals. All pigs were fed with normal pig diet. Blood and tissue samples were collected every 6 months.

### Stereomicroscopy and endoscopy

Macroscopic images of colonic lesions were taken *in vivo* with a STORZ colonoscopy system and *ex vivo* with a Zeiss Stemi 11 stereomicroscope. Colonoscopy examination was carried out approximately every 6 months, starting at 3 months old. Polyp and normal mucosa biopsy samples were divided and portions were fixed as described below, frozen in 2-methylbutane (OCT) for cryosection, and snap frozen and stored at −80 °C for molecular analyses.

### Histochemistry

For histopathology analysis, specimens were fixed in 4% buffered paraformaldehyde, embedded in paraffin, sectioned (3 μm) and stained with haematoxilin and eosin (H&E). Polyps were classified as LG-IEN or HG-IEN according to the AJCC TNM staging system^32^.

For immunohistochemistry, specimens were fixed using the HOPE technique^33^, embedded in paraffin and 3 μm sections cut. Sections were dewaxed, microwaved (10 min, 600 watt), and incubated with mouse anti-porcine primary antibodies: CD3 (1:100, clone BB23-8E6), CD4 (1:100, clone 74-12-4), CD8 (1:100, clone 76-2-11). All primary antibodies were obtained from SouthernBiotech. Tissue sections were incubated with biotinylated anti-mouse IgG (1:200, Vector Laboratories) followed by Elite ABC kit incubation. Antibody binding was detected with a DAB Peroxidase Substrate kit (Vector Laboratories).

### Laser microdissection

Laser microdissection (LMD) of cryosectioned samples was performed immediately after H&E staining using a Leica Microsystems Laser Microdissection Systems 6000 and Leica Application Suite software (Leica). In total, ten LMD captured crypts per polyp sample were cut, providing 300–500 cells, and collected into 50 µL lysis buffer from the AllPrep® DNA/RNA Micro Kit (Qiagen), and a further 50 µL lysis buffer added after dissection. Dissected samples were stored at −80 °C. Genomic DNA and total RNA from the same sample were isolated using the AllPrep® DNA/RNA Micro Kit according to the manufacturer’s instructions (Qiagen).

### Whole transcriptome amplification of LMD samples

For allele expression assessment by pyrosequencing, 100 pg total RNA isolated from LMD samples was used for whole transcriptome amplification using the QuantiTect Whole Trancriptome Kit according to the manufacturer’s instructions (Qiagen).

### Library preparation

Libraries for RNA sequencing of whole biopsies were prepared using the TruSeq Stranded mRNA LT Kit (Illumina). RNA integrity and fragment size were tested using the RNA6000 Nano kit (Agilent) on an Agilent Bioanalyzer 2100 (Agilent).

Libraries for RNA sequencing of LMD samples were prepared using SMART-Seq. 2 Ultra-low kit (Clontech) and Nextera XT DNA SMP preparation kit according to the manufacturers protocol (Illumina). RNA and dscDNA integrity and fragment size were tested using RNA6000 Pico and High Sensitivity DNA kits (Agilent).

### NGS RNA sequencing

Libraries were sequenced with a HiSeq. 2500 ultra-high-throughput sequencing system (Illumina) to produce 100-base-paired end reads. An average of 45 million reads per sample were generated and mapped to the porcine reference genome (Sscrofa10.2) using the 2-pass method of the *STAR* aligner with default parameters^34^. 80% of these reads uniquely hit to the reference genome. Duplicate reads were denoted with the *MarkDuplicates* tool of Picard (http://broadinstitute.github.io/picard). Aligned reads (omitting duplicate reads) were assigned to gene sequences as defined in the 10.2.77 porcine gene set and counted with *featureCounts* ^35^. Normalisation of read counts and estimation of fold change was carried out using *DESeq. 2* ^36^. Hierarchical clusters and heat maps for 60 genes with the most significantly different levels of expression were generated using the *python* (http://www.python.org) packages *spatial* and *cluster* of *scipy* ^37^ along with *matplotlib* ^38^.

Variant calling based on *STAR* alignments was performed according to GATK^39^ best practice recommendations for RNAseq^40, 41^. The GATK tool *SplitNCigarReads* was used to split reads into exons and remove false variants resulting from overhangs. This step included reassignment of the *STAR* alignment mapping qualities. GATK recalibration of base scores was based on the Ensembl release 83 variant database. Variant calling was carried out using GATK *HaplotypeCaller* with the *dontUsedSoftClippedBases* option. GATK *VariantFiltration* was applied to clusters of at least 3 SNPs within a window of 35 bases between them with the following parameters: Fisher strand value (FS) >30.0 and a quality by depth value (QD) <2.0. The probability of allelic imbalance for each SNP was calculated based on the number of reference and alternate allele reads in heterozygous animals using a two-sided binomial test. P values were adjusted for false discovery rate (q value) to take account of multiple testing.

### Gene set enrichment analysis

Gene expression data (HTSeq-counts) for stage I human colon cancers (n = 80) and normal samples of colonic mucosa (n = 41) were retrieved from The Cancer Genome Atlas (TCGA) using the R/Bioconductor package TCGAbiolinks (version 2.2.3). From the TCGA, we also retrieved gene expression data (HTSeq-counts) for stage I microsatellite stability (MSS) (n = 23) and stage I low level microsatellite instability (MSI-L) (n = 11) human colon cancers. Log2 fold change of gene expression was analysed using the included functions *TCGAanalyze_DEA* and *TCGAanalyze_LevelTab*. For porcine colon polyps, the numbers of mapped reads to each gene were counted using the *featureCount* tool implemented in the subread package (version 1.5.0-p2)^35^. The gene annotation file was downloaded from Ensembl (Ensembl release 86). Log2 fold change of gene expression (HG-IEN versus LG-IEN) was analysed using the R/Bioconductor package DESeq. 2 (version 1.14.0)^36^.

Gene set enrichment analysis^42^ was performed using Human Genome Organization (HUGO) gene symbols together with log2 fold change of gene expression as input. The tool GSEAPreranked implemented in GSEA (version 2.2.3) was used to estimate gene set enrichment with the following parameters: classic enrichment statistics, 1000 permutations, and hallmark gene sets collection from Molecular Signatures Database (version 5.2)^43^. P values were adjusted for false discovery rate (q value) to take account of multiple testing.

PANTHER Gene List Analysis^44^ was used to perform functional pathway analysis for the top >300 differentially expressed genes with PANTHER pathways as annotation data set and statistical overrepresentation test with default settings.

### Quantitative RT-PCR

Total RNA was extracted using Direct-zol RNA Mini Prep Kit (Zymo Research) according to the manufacturer’s instructions. 200 ng total RNA was used to synthesise complementary DNA (cDNA) using Superscript IV reverse transcriptase (Invitrogen). Two-step qPCR experiments were performed using Fast SybrGreen MasterMix (Applied Biosystems) and run on an ABI 7500 thermocycler (Applied Biosystems). Primer specificity and capture temperature were determined by melt curve analysis. The relative expression difference between the groups in all tissues was calculated for each sample (ΔΔCT). All cDNA samples were assayed in triplicate and relative expression levels normalised to *PPIA* and *RPS28* reference genes. Reference genes were selected from a set of eight examined genes *ATP6*, *RPS28*, *GAPDH*, *HPRT1*, *RPS23*, *TPT1*, *PPIA*, *RN18S* based on stability values from the NormFinder.

### Allele quantification using pyrosequencing

Genomic DNA was extracted using the GenElute Mammalian Genomic DNA Kit (Sigma Aldrich) and used for allele quantification in heterozygous SNPs. The allele proportion of 50:50 on DNA level for valid pyrosequencing assays was expected. Total RNA was extracted using the Direct-zol RNA Mini Prep Kit (Zymo Research) according to the manufacturer’s instructions. 200 ng total RNA was used to synthesise complementary DNA (cDNA) using Superscript IV reverse transcriptase (Invitrogen). cDNA was amplified by RT-PCR, and allele expression for selected SNPs was analysed by pyrosequencing using the Pyromark Q24 system (Qiagen). Pyrosequencing primers were designed using the PyroMark Assay Design Software 2.0 (Qiagen). Allele expression differences at individual SNPs were evaluated using Students t-test.

### Primers

The authors will provide all primer sequences used in this study on request.

## Electronic supplementary material

Supplementary information
