# Supplementary material for: Porcine familial adenomatous polyposis model enables systematic analysis of early events in adenoma progression
Source: Sci Rep. 2017 Jul 26;7:6613. doi: 10.1038/s41598-017-06741-8 (PMC5529429; doi:10.1038/s41598-017-06741-8)
Supplement: Supplementary file 1 — Supplementary information [file 41598_2017_6741_MOESM1_ESM.docx]

Supplementary information

**Porcine familial adenomatous polyposis model enables systematic analysis of early events in adenoma progression**

Tatiana Flisikowska^1^, Monika Stachowiak^2^, Hongen Xu^3^, Alexandra Wagner^1^, Alejandra Hernandez-Caceres^1^, Christine Wurmser^4^, Carolin Wander^1^, Hubert Pausch^4^, Anna Perkowska^2^, Konrad Fischer^1^, Dmitrij Frishman^3,5,6^, Ruedi Fries^4^, Marek Switonski^2^, Alexander Kind^1^, Dieter Saur^7^, Angelika Schnieke^1^, Krzysztof Flisikowski^1^

**Author Affiliations**

1. Chair of Livestock Biotechnology, Technische Universität München, Freising, Germany.

2. Department of Genetics and Animal Breeding, Poznan University of Life Sciences, Poznan, Poland.

3. Department of Bioinformatics, Wissenschaftszentrum Weihenstephan, Technische Universität München, Freising, Germany.

4. Chair of Animal Breeding, Technische Universität München, Freising, Germany.

5. Institute of Bioinformatics and Systems Biology, Helmholtz Zentrum Munich - German Research Center for Environmental Health, Neuherberg, Germany.

6. St Petersburg State Polytechnic University, St Petersburg, Russia.

7. Klinikum Rechts der Isar II, Technische Universität München, Munich, Germany.

**Supporting Information - Description**

**Supplementary Table 1.** Genes showing differential expression in whole samples from porcine polyps histologically classified as LG-IEN or HG-IEN (Excel file). Data with adjusted P value <0.05 is shown.

**Supplementary Table 2.** qPCR validation of expression of selected genes in porcine LG-IEN and HG- IEN polyps.

**Supplementary Table 3.** Genes showing differential expression in microdissected porcine LG-IEN and HG-IEN adenoma from the same polyps (Excel file). Data with P value <0.05 is shown.

**Supplementary Table 4.** qPCR validation of top 5 genes differentially expressed in microdissected LG-IEN (n=20) and HG-IEN (n=20) samples.

**Supplementary Table 5.** SNPs showing allelic imbalance in porcine LG-IEN and HG-IEN whole samples (Excel file). Variant calling revealed 705,000 heterozygous SNPs, of which 48,000 (6%) showed allelic imbalance in at least one sample either in LG-IEN and HG-IEN polyps (*PvalueAdj* < 0.05). Imbalanced allele expression was detected for SNPs in several cancer-related genes such as *EpCAM*, *MSH2*, *MMP7*, *MMP12*, *PLOR1D* and *CCL5.* Data with adjusted P value < 7x10^-11^ is shown.

**Supplementary Figure 1.** H&E stained sections of biopsies from the tumour indicated by the arrow in Figure 1a. Arrows in panel (f) indicate a blood vessel with erythrocytes; the arrowhead marks suspected vessel infiltration by a tumour cell. Panels (d) and (f) show indicated high magnification details of panels (c) and (e), respectively. Scale bars, 50µm.

**Supplementary Figure 2.** Heatmap showing unsupervised clustering of histologically unclassified 1 cm diameter polyps taken from 22 *APC^1311/+^* pigs. The top 60 differentially expressed genes correlated with severity of polyposis (LP- low polyp number vs HP- high polyp number) in the source animals.

**Supplementary Figure 3**. T cell infiltration of adenomatous polyp. (a) Immunohistochemical staining of CD3^+^ (brown) infiltrating T cells (a, b). Panel (a) shows high magnification details of panel a. Panel c shows CD4^+^ (brown) and panel d CD8^+^ (brown) infiltrating T cells. IEN, high-grade intraepithelial neoplasia.

**Supplementary Table 1.** Genes showing differential expression in whole samples from porcine polyps histologically classified as LG-IEN or HG-IEN (Excel file). Data with adjusted P value <0.05 is shown.

| Id | ensGeneId | geneSymbol | log2FoldChange | pValue | adjustedPvalue |
| --- | --- | --- | --- | --- | --- |
| 104 | ENSSSCG00000021273 | SYNE1 | 1,472829724 | 5,82001E-08 | 0,000419186 |
| 4030 | ENSSSCG00000027969 | AHNAK | 1,686111121 | 4,03693E-08 | 0,000419186 |
| 15772 | ENSSSCG00000006530 | EFNA1 | -0,717619075 | 4,04085E-07 | 0,00194028 |
| 8088 | ENSSSCG00000015853 | HERC2 | 1,13331256 | 5,70526E-07 | 0,002054608 |
| 25103 | ENSSSCG00000029160 | HSPA1L | -1,536457864 | 1,72348E-06 | 0,004965354 |
| 611 | ENSSSCG00000004520 | MAPK4 | 1,516004246 | 2,79723E-06 | 0,006715693 |
| 4056 | ENSSSCG00000013087 | DAK | -0,393444182 | 3,29644E-06 | 0,006783609 |
| 23033 | ENSSSCG00000001917 | CD276 | -0,844508726 | 4,57106E-06 | 0,00823077 |
| 1956 | ENSSSCG00000005615 | PTRH1 | -0,945301305 | 7,80082E-06 | 0,010215532 |
| 4270 | ENSSSCG00000013252 | F2 | -1,439199721 | 7,10441E-06 | 0,010215532 |
| 15363 | ENSSSCG00000006161 | IL7 | 1,0939743 | 6,88349E-06 | 0,010215532 |
| 3455 | ENSSSCG00000011947 | ZPLD1 | -1,327239008 | 1,47116E-05 | 0,013355526 |
| 8573 | ENSSSCG00000021610 | CHPF | -0,639641258 | 1,1585E-05 | 0,013355526 |
| 17285 | ENSSSCG00000002013 | DHRS4 | -0,557421281 | 1,40455E-05 | 0,013355526 |
| 17474 | ENSSSCG00000002243 | SLC12A6 | 1,032033995 | 1,45863E-05 | 0,013355526 |
| 21288 | ENSSSCG00000017227 | FDXR | -0,540264467 | 1,48343E-05 | 0,013355526 |
| 16661 | ENSSSCG00000001418 | NEU1 | -0,841689087 | 2,05518E-05 | 0,015581483 |
| 19468 | ENSSSCG00000009555 | LAMP1 | -0,39073283 | 1,95407E-05 | 0,015581483 |
| 23651 | ENSSSCG00000028391 | PPP1R13L | -0,896064853 | 2,00209E-05 | 0,015581483 |
| 25267 | ENSSSCG00000021730 | RNH1 | -0,567085816 | 2,56728E-05 | 0,018490805 |
| 7803 | ENSSSCG00000026635 | SLC2A1 | -0,769590477 | 2,92447E-05 | 0,020060487 |
| 1721 | ENSSSCG00000005423 | ABCA1 | 1,066931778 | 3,32654E-05 | 0,020834263 |
| 8898 | ENSSSCG00000009673 | NUGGC | 1,347462744 | 3,27181E-05 | 0,020834263 |
| 19573 | ENSSSCG00000016833 | CAPSL | -0,842953048 | 3,53457E-05 | 0,021214769 |
| 801 | ENSSSCG00000004674 | SHF | -0,997911092 | 5,13736E-05 | 0,02590067 |
| 7597 | ENSSSCG00000003802 | SLC35D1 | 1,013500403 | 5,2143E-05 | 0,02590067 |
| 19079 | ENSSSCG00000025308 | IL17D | -0,510852908 | 4,97491E-05 | 0,02590067 |
| 20124 | ENSSSCG00000030694 | PKP1 | 1,292312719 | 5,02275E-05 | 0,02590067 |
| 21192 | ENSSSCG00000017148 | RNF213 | 1,182687088 | 5,00245E-05 | 0,02590067 |
| 18044 | ENSSSCG00000021663 | NOL12 | -0,980237211 | 5,63083E-05 | 0,027037369 |
| 6974 | ENSSSCG00000003401 | KIF1B | 0,821116806 | 5,83813E-05 | 0,027128456 |
| 19629 | ENSSSCG00000030304 | NNT | 0,707232303 | 6,25387E-05 | 0,028152188 |
| 2462 | ENSSSCG00000011332 | SETD2 | 0,288039759 | 7,12388E-05 | 0,029319871 |
| 2682 | ENSSSCG00000011493 | ATXN7 | 0,364014063 | 7,02414E-05 | 0,029319871 |
| 22303 | ENSSSCG00000022033 | SLC46A1 | -1,037851138 | 6,94894E-05 | 0,029319871 |
| 22325 | ENSSSCG00000016416 | SHH | -1,237069637 | 7,54694E-05 | 0,030198252 |
| 21398 | ENSSSCG00000017316 | NSF | 0,528357221 | 8,1415E-05 | 0,031133759 |
| 22341 | ENSSSCG00000020663 | KMT2C | 0,932819968 | 8,213E-05 | 0,031133759 |
| 210 | ENSSSCG00000004179 | VNN2 | -1,2303979 | 8,6786E-05 | 0,031253811 |
| 2697 | ENSSSCG00000020842 | TAF13 | 1,180232154 | 8,67854E-05 | 0,031253811 |
| 7806 | ENSSSCG00000027330 | SLC2A1 | -0,765524118 | 8,97801E-05 | 0,031543466 |
| 17211 | ENSSSCG00000001963 | EGLN3 | -1,178934849 | 9,26788E-05 | 0,031786614 |
| 21440 | ENSSSCG00000017355 | GRN | -0,734461237 | 0,000105456 | 0,035327683 |
| 1615 | ENSSSCG00000005325 | TMEM8B | -0,75458121 | 0,000118464 | 0,038783506 |
| 22367 | ENSSSCG00000024431 | ATG9B | -1,217752171 | 0,000122689 | 0,03927406 |
| 2009 | ENSSSCG00000005673 | PSEUDOGENE | -1,083116995 | 0,000129474 | 0,040545096 |
| 15089 | ENSSSCG00000005946 | WISP1 | -1,196839627 | 0,000135722 | 0,04159745 |
| 9928 | ENSSSCG00000010579 | GBF1 | 0,350353224 | 0,00013917 | 0,041765414 |
| 13017 | ENSSSCG00000008022 | TELO2 | -0,43994936 | 0,000166694 | 0,047656121 |
| 17043 | ENSSSCG00000001799 | HDGFRP3 | 0,961711444 | 0,000168724 | 0,047656121 |
| 22398 | ENSSSCG00000016472 | KEL | -1,177907209 | 0,000167439 | 0,047656121 |
| 4918 | ENSSSCG00000013616 | ZNF653 | -0,482312715 | 0,000177155 | 0,049075256 |
| 9141 | ENSSSCG00000025579 | UNKNOWN | 1,087262006 | 0,00018642 | 0,050667421 |
| 12860 | ENSSSCG00000022149 | RMI2 | -0,96272494 | 0,000213997 | 0,055046796 |
| 14524 | ENSSSCG00000030787 | CH242-51E15.1 | -1,169090889 | 0,000212698 | 0,055046796 |
| 15058 | ENSSSCG00000006957 | RHPN1 | -1,112905576 | 0,000207364 | 0,055046796 |
| 2570 | ENSSSCG00000011424 | TEX264 | -0,381654569 | 0,000254902 | 0,056087961 |
| 7108 | ENSSSCG00000025146 | HSPG2 | -0,805913367 | 0,000251452 | 0,056087961 |
| 10443 | ENSSSCG00000014831 | PAAF1 | -0,622524303 | 0,00023404 | 0,056087961 |
| 10643 | ENSSSCG00000014983 | MMP20 | -1,135127908 | 0,000239911 | 0,056087961 |
| 12286 | ENSSSCG00000009157 | TET2 | 0,694754463 | 0,000254138 | 0,056087961 |
| 12332 | ENSSSCG00000022353 | RAP1GDS1 | 0,2769484 | 0,000241188 | 0,056087961 |
| 12834 | ENSSSCG00000007872 | XYLT1 | 0,890703232 | 0,000262946 | 0,056087961 |
| 15824 | ENSSSCG00000006577 | S100A5 | -1,160022816 | 0,000223267 | 0,056087961 |
| 15966 | ENSSSCG00000024481 | PDE4DIP | 0,901629128 | 0,000264208 | 0,056087961 |
| 17378 | ENSSSCG00000002127 | CHD8 | 0,31465704 | 0,000239923 | 0,056087961 |
| 19374 | ENSSSCG00000009498 | ABCC4 | 0,929586466 | 0,000264768 | 0,056087961 |
| 25096 | ENSSSCG00000018091 | MT-ND5 | 0,267700345 | 0,000247659 | 0,056087961 |
| 24296 | ENSSSCG00000023114 | PDP2 | 0,529369295 | 0,00027962 | 0,058375668 |
| 21942 | ENSSSCG00000017764 | KIAA0100 | 0,339642174 | 0,000293797 | 0,060459153 |
| 25291 | ENSSSCG00000027580 | UNKNOWN | -1,03978764 | 0,000317212 | 0,064358234 |
| 20187 | ENSSSCG00000010960 | SLC28A3 | -0,755721216 | 0,000326579 | 0,065338511 |
| 3697 | ENSSSCG00000022568 | TMPRSS3 | -0,900324409 | 0,000361238 | 0,070319464 |
| 18556 | ENSSSCG00000000539 | TMTC1 | 0,665008057 | 0,000357145 | 0,070319464 |
| 795 | ENSSSCG00000004668 | SLC30A4 | 0,968410548 | 0,000383373 | 0,072650999 |
| 3351 | ENSSSCG00000025436 | ILDR1 | -0,939744827 | 0,000398668 | 0,072650999 |
| 6530 | ENSSSCG00000003107 | ARHGAP35 | 0,273794044 | 0,000432981 | 0,072650999 |
| 6901 | ENSSSCG00000021073 | UNKNOWN | -1,075720509 | 0,000400028 | 0,072650999 |
| 9328 | ENSSSCG00000010067 | MIF | -0,58205096 | 0,000413093 | 0,072650999 |
| 11391 | ENSSSCG00000015487 | TNFSF18 | -1,121589825 | 0,000428499 | 0,072650999 |
| 12484 | ENSSSCG00000027768 | SLC29A4 | -0,966668998 | 0,000433737 | 0,072650999 |
| 13170 | ENSSSCG00000023884 | UNKNOWN | 1,118111806 | 0,000427222 | 0,072650999 |
| 16001 | ENSSSCG00000006734 | CD101 | 0,957797865 | 0,000390662 | 0,072650999 |
| 17717 | ENSSSCG00000002472 | IFI27L2 | -0,596111586 | 0,000431879 | 0,072650999 |
| 20532 | ENSSSCG00000007023 | KAT6A | 0,514113209 | 0,000412809 | 0,072650999 |
| 22277 | ENSSSCG00000018034 | CENPV | -0,494312689 | 0,000398806 | 0,072650999 |
| 23014 | ENSSSCG00000030658 | SERPINF2 | -0,75474103 | 0,000449106 | 0,074360656 |
| 2178 | ENSSSCG00000005830 | CCDC183 | -0,978635687 | 0,000456454 | 0,074416556 |
| 8370 | ENSSSCG00000016074 | ANKRD44 | 0,635192374 | 0,000459776 | 0,074416556 |
| 807 | ENSSSCG00000004679 | SORD | -0,583566891 | 0,000498253 | 0,077670808 |
| 4850 | ENSSSCG00000013671 | ZNF426 | 0,733417199 | 0,00050145 | 0,077670808 |
| 11628 | ENSSSCG00000008701 | LREAP1 | -0,409544559 | 0,000498548 | 0,077670808 |
| 16696 | ENSSSCG00000030750 | SLA-DQB2 | 1,035683652 | 0,000488854 | 0,077670808 |
| 3928 | ENSSSCG00000013004 | TM7SF2 | -0,915694926 | 0,000527608 | 0,08000203 |
| 12920 | ENSSSCG00000025561 | VASN | -0,845973316 | 0,00052517 | 0,08000203 |
| 21417 | ENSSSCG00000017338 | PLCD3 | -0,765774961 | 0,000539526 | 0,080957008 |
| 3734 | ENSSSCG00000012088 | POFUT2 | -0,537997239 | 0,00054515 | 0,080957572 |
| 5968 | ENSSSCG00000002688 | PLCG2 | 0,596083581 | 0,000551237 | 0,081026186 |
| 2751 | ENSSSCG00000023437 | ITPR1 | 0,837994583 | 0,000575191 | 0,083688382 |
| 12781 | ENSSSCG00000025037 | PRKCB | 1,028153861 | 0,000580968 | 0,083688382 |
| 830 | ENSSSCG00000004699 | ELL3 | 1,087084115 | 0,000639775 | 0,084313356 |
| 2936 | ENSSSCG00000027164 |  | -0,618761397 | 0,000632227 | 0,084313356 |
| 3735 | ENSSSCG00000028846 |  | -0,70279238 | 0,000595397 | 0,084313356 |
| 4238 | ENSSSCG00000023418 |  | 0,447484933 | 0,000641007 | 0,084313356 |
| 4639 | ENSSSCG00000013858 | CALR3 | -0,931384051 | 0,000618079 | 0,084313356 |
| 6158 | ENSSSCG00000030300 | MT2A | -0,966284178 | 0,000650116 | 0,084313356 |
| 8720 | ENSSSCG00000016342 | HES6 | -0,673913543 | 0,000623956 | 0,084313356 |
| 9335 | ENSSSCG00000010075 | VPREB3 | 1,100330268 | 0,000612954 | 0,084313356 |
| 11555 | ENSSSCG00000015617 | G0S2 | -1,092374015 | 0,000656622 | 0,084313356 |
| 20119 | ENSSSCG00000027928 | TMEM9 | -0,752676724 | 0,000605965 | 0,084313356 |
| 22418 | ENSSSCG00000016487 |  | 0,95070706 | 0,000649767 | 0,084313356 |
| 24057 | ENSSSCG00000027115 |  | 0,480721108 | 0,000661396 | 0,084313356 |
| 25302 | ENSSSCG00000030700 | DBN1 | -1,013475582 | 0,000641578 | 0,084313356 |
| 2759 | ENSSSCG00000011538 | LMCD1 | -0,574815827 | 0,000690953 | 0,087308629 |
| 21199 | ENSSSCG00000017156 |  | -0,443143097 | 0,0007065 | 0,088496787 |
| 12900 | ENSSSCG00000007925 | NAGPA | -0,403941015 | 0,000720413 | 0,089051548 |
| 15410 | ENSSSCG00000006201 | ARFGEF1 | 0,318945947 | 0,000723293 | 0,089051548 |
| 4876 | ENSSSCG00000013651 | FDX1L | -0,381166976 | 0,00074044 | 0,09039014 |
| 6146 | ENSSSCG00000029578 | NFATC3 | 0,451598243 | 0,000763533 | 0,090712544 |
| 6669 | ENSSSCG00000026561 | CLEC11A | -0,699309113 | 0,000764727 | 0,090712544 |
| 15609 | ENSSSCG00000028374 |  | 1,053260806 | 0,000759719 | 0,090712544 |
| 21491 | ENSSSCG00000017393 | CNTNAP1 | 0,790507907 | 0,00076827 | 0,090712544 |
| 19072 | ENSSSCG00000000982 | TTLL8 | -0,893603164 | 0,000778013 | 0,091116082 |
| 25296 | ENSSSCG00000027350 | DPP7 | -0,40703251 | 0,000806671 | 0,093710485 |
| 18956 | ENSSSCG00000000884 |  | 0,444486917 | 0,000816937 | 0,094143802 |
| 14385 | ENSSSCG00000012394 | GJB1 | -0,528195932 | 0,000842975 | 0,096373463 |
| 15696 | ENSSSCG00000026210 | FCRL5 | 1,080173865 | 0,000863328 | 0,096805245 |
| 16142 | ENSSSCG00000006857 | COL11A1 | -0,926004765 | 0,000866913 | 0,096805245 |
| 21583 | ENSSSCG00000017494 | IKZF3 | 1,047274102 | 0,000855956 | 0,096805245 |
| 6783 | ENSSSCG00000003288 | HSPBP1 | -0,442629186 | 0,000889996 | 0,098618451 |
| 10790 | ENSSSCG00000023324 | KMT2A | 0,74402904 | 0,000931734 | 0,10245515 |

| Gene symbol | Gene description | RNA seq  Fold change  LG vs HG | *P* value | qPCR  Fold change  LG vs HG | *P* value |
| --- | --- | --- | --- | --- | --- |
| *IL7* | Interleukin 7 | -1.09 | 6.88x10^-6^ | -5.18 | 4.02x10^-7^ |
| *EBF1* | Early B-cell factor 1 | -1.69 | 7.33x10^-10^ | -4.03 | 0.008 |
| *CD40-1* | CD40 molecule isoform 1 | -1.10 | 2.09x10^-12^ | -1.96 | 0.013 |
| *CD40-2* | CD40 molecule isoform 2 | -1.22 | 2.88x10^-5^ | -2.04 | 0.085 |
| *CD40-LG* | CD40 ligand | -1.28 | 4.56x10^-6^ | -2.11 | 0.009 |
| *TRAF5* | TNF receptor associated factor 5 | -1.16 | 1.20x10^-9^ | -2.21 | 0.007 |
| *IL21* | Interleukin 21 | -2.30 | 2.06x10^-17^ | -8.97 | 0.006 |
| *CD101* | CD101 molecule | -0.95 | 0.0003 | -3.08 | 0.044 |
| *S100A8* | S100 calcium-binding protein A8 | 2.79 | 3.16x10^-12^ | 3.79 | 0.021 |
| *S100A9* | S100 calcium-binding protein A9 | -2.34 | 5.27x10^-9^ | -2.66 | 0.048 |
| *IL20RA* | Interleukin 20 receptor subunit alpha | 2.18 | 6.08x10^-11^ | 3.40 | 6.7x10^-6^ |

**Supplementary Table 2.** qPCR validation of selected genes in the low grade (LG- IEN) and high grade intraepithelial (HG- IEN) polyps.

**Supplementary Table 3.** Genes showing differential expression in microdissected porcine LG-IEN and HG-IEN adenoma from the same polyps (Excel file). Data with P value <0.05 is shown.

| ID | ensGeneId | geneSymbol | log2FoldChange | pvalue |
| --- | --- | --- | --- | --- |
| 2820 | ENSSSCG00000011592 | PLXND1 | 2,26 | 5,40E-08 |
| 11541 | ENSSSCG00000024509 | SLC30A1 | 2,21 | 9,74E-08 |
| 7280 | ENSSSCG00000022120 |  | -1,77 | 2,08E-06 |
| 16225 | ENSSSCG00000030801 | GBP6 | 1,91 | 3,07E-06 |
| 17624 | ENSSSCG00000002389 | VASH1 | 1,60 | 7,65E-05 |
| 20005 | ENSSSCG00000010833 | TAF1A | 1,25 | 6,42E-05 |
| 20019 | ENSSSCG00000010840 | RGS7 | -1,43 | 8,70E-05 |
| 1237 | ENSSSCG00000005023 | ABHD12B | -1,57 | 1,40E-04 |
| 12475 | ENSSSCG00000007567 | CHST12 | -1,54 | 1,63E-04 |
| 15072 | ENSSSCG00000005934 | TRAPPC9 | 1,04 | 2,30E-04 |
| 1905 | ENSSSCG00000023197 |  | 1,38 | 3,44E-04 |
| 16159 | ENSSSCG00000006872 | AGL | -1,20 | 3,91E-04 |
| 21735 | ENSSSCG00000017613 | COIL | 1,33 | 4,52E-04 |
| 1735 | ENSSSCG00000005436 | RAD23B | -0,77 | 5,83E-04 |
| 22350 | ENSSSCG00000016435 | SMARCD3 | 1,32 | 6,79E-04 |
| 1848 | ENSSSCG00000030089 | RBM18 | 1,05 | 7,97E-04 |
| 9739 | ENSSSCG00000023931 |  | -1,30 | 8,36E-04 |
| 17269 | ENSSSCG00000001997 |  | -1,21 | 7,73E-04 |
| 136 | ENSSSCG00000004114 | ADGB | 1,29 | 9,66E-04 |
| 2617 | ENSSSCG00000011452 |  | 1,31 | 9,45E-04 |
| 7299 | ENSSSCG00000025344 | PHC2 | -0,82 | 9,92E-04 |
| 8893 | ENSSSCG00000020846 | SCARA3 | 1,27 | 1,38E-03 |
| 11450 | ENSSSCG00000015531 |  | 1,20 | 1,33E-03 |
| 794 | ENSSSCG00000030710 | PLDN | 1,26 | 1,51E-03 |
| 10166 | ENSSSCG00000014578 | DENND5A | -1,24 | 1,45E-03 |
| 24559 | ENSSSCG00000027181 |  | 0,90 | 1,59E-03 |

**Supplementary Table 4.** qPCR validation of top 5 genes differentially expressed in microdissected LG-IEN (n=20) and HG-IEN (n=20) samples.

| Gene symbol | Gene description | RNA seq  Fold change  HG vs LG | *P* value | qPCR  Fold change  HG vs LG | *P* value |
| --- | --- | --- | --- | --- | --- |
| *PLXND1* | plexin D1 | 2.26 | 5.40x10^-8^ | 4.23 | 1.40x10^-3^ |
| *SLC30A1* | solute carrier family 30 member 1 | 2.21 | 9.74x10^-8^ | 3.95 | 0.0009 |
| *GBP6* | guanylate binding protein family member 6 | 1.91 | 3.07x10^-6^ | 5.40 | 2.10x10^-3^ |
| *VASH1* | vasohibin 1 | 1.60 | 7.65x10^-5^ | 3.10 | 0.0091 |
| *SMARCD3* | SWI/SNF related, matrix associated, actin dependent regulator of chromatin, subfamily d, member 3 | 1.32 | 6.79x10^-4^ | 2.88 | 1.99x10^-3^ |

**Supplementary Table 5.** SNPs showing allelic imbalance in porcine LG-IEN and HG-IEN whole samples (Excel file). Variant calling revealed 705,000 heterozygous SNPs, of which 48,000 (6%) showed allelic imbalance in at least one sample either in LG-IEN and HG-IEN polyps (*PvalueAdj* < 0.05). Imbalanced allele expression was detected for SNPs in several cancer-related genes such as *EpCAM*, *MSH2*, *MMP7*, *MMP12*, *PLOR1D* and *CCL5.* Data with adjusted P value < 7x10^-11^ is shown.

|  | chr | pos | gene | n_ref | n_alt | p-value | p-value-adj |
| --- | --- | --- | --- | --- | --- | --- | --- |
| 3_59628700 | 3 | 59628700 |  | 199 | 1821 | 0,00E+00 | 0,00E+00 |
| 6_45194986 | 6 | 45194986 |  | 578 | 2697 | 0,00E+00 | 0,00E+00 |
| 6_45974081 | 6 | 45974081 |  | 310 | 2126 | 0,00E+00 | 0,00E+00 |
| 3_59628709 | 3 | 59628709 |  | 202 | 2072 | 0,00E+00 | 0,00E+00 |
| 2_141861499 | 2 | 141861499 |  | 340 | 1650 | 7,82E-206 | 8,19E-202 |
| 3_99311113 | 3 | 99311113 |  | 231 | 1267 | 4,03E-173 | 3,51E-169 |
| 4_116884972 | 4 | 116884972 | BCL2L15 | 1723 | 471 | 7,20E-167 | 5,38E-163 |
| 2_146230334 | 2 | 146230334 |  | 1525 | 419 | 4,09E-147 | 2,68E-143 |
| 12_41228440 | 12 | 41228440 | CCL5 | 1016 | 181 | 2,49E-141 | 1,45E-137 |
| 6_52531857 | 6 | 52531857 |  | 1795 | 595 | 5,99E-139 | 3,14E-135 |
| 7_24749736 | 7 | 24749736 |  | 179 | 985 | 2,97E-135 | 1,42E-131 |
| 8_115973143 | 8 | 115973143 | UGT8 | 163 | 944 | 3,83E-134 | 1,67E-130 |
| 4_116885819 | 4 | 116885819 | BCL2L15 | 1791 | 614 | 1,35E-132 | 5,42E-129 |
| 5_36185493 | 5 | 36185493 |  | 76 | 708 | 2,53E-129 | 9,46E-126 |
| 13_145010795 | 13 | 145010795 | MUC13A | 861 | 2151 | 6,18E-126 | 2,16E-122 |
| 2_85431247 | 2 | 85431247 | NSA2 | 2327 | 982 | 3,16E-124 | 1,03E-120 |
| 4_116885717 | 4 | 116885717 | BCL2L15 | 1721 | 603 | 9,83E-124 | 3,03E-120 |
| 11_4679598 | 11 | 4679598 | POLR1D | 184 | 931 | 1,41E-120 | 4,09E-117 |
| 4_139601552 | 4 | 139601552 |  | 1173 | 313 | 5,46E-117 | 1,50E-113 |
| 11_4679569 | 11 | 4679569 | POLR1D | 237 | 1022 | 2,16E-116 | 5,67E-113 |
| 8_75412057 | 8 | 75412057 |  | 251 | 1031 | 1,88E-112 | 4,69E-109 |
| 4_61946993 | 4 | 61946993 |  | 1423 | 473 | 4,08E-110 | 9,71E-107 |
| 2_121507509 | 2 | 121507509 | REEP5 | 536 | 1529 | 4,80E-110 | 1,09E-106 |
| 3_99312442 | 3 | 99312442 |  | 131 | 762 | 6,88E-109 | 1,50E-105 |
| 7_5314927 | 7 | 5314927 | BMP6 | 1432 | 486 | 8,20E-108 | 1,65E-104 |
| 4_61946994 | 4 | 61946994 |  | 1410 | 473 | 7,94E-108 | 1,66E-104 |
| 4_139601449 | 4 | 139601449 |  | 1091 | 299 | 5,27E-106 | 1,02E-102 |
| 3_99312370 | 3 | 99312370 | EPCAM | 166 | 826 | 7,64E-106 | 1,43E-102 |
| 1_32266392 | 1 | 32266392 |  | 399 | 1266 | 6,24E-105 | 1,13E-101 |
| 12_25313351 | 12 | 25313351 |  | 202 | 895 | 1,65E-104 | 2,88E-101 |
| 12_25352132 | 12 | 25352132 |  | 124 | 725 | 5,12E-104 | 8,65E-101 |
| 4_116885869 | 4 | 116885869 | BCL2L15 | 1477 | 528 | 1,77E-103 | 2,89E-100 |
| 3_99311854 | 3 | 99311854 |  | 250 | 985 | 1,98E-103 | 3,13E-100 |
| 13_145010793 | 13 | 145010793 | MUC13A | 965 | 2151 | 1,62E-102 | 2,50E-99 |
| 4_139601462 | 4 | 139601462 |  | 1052 | 289 | 5,06E-102 | 7,56E-99 |
| 5_36180004 | 5 | 36180004 |  | 66 | 570 | 4,72E-101 | 6,86E-98 |
| 2_61156235 | 2 | 61156235 |  | 199 | 859 | 3,60E-98 | 5,09E-95 |
| 4_139601578 | 4 | 139601578 |  | 849 | 201 | 3,13E-95 | 4,31E-92 |
| 2_146230312 | 2 | 146230312 |  | 1466 | 558 | 1,31E-93 | 1,76E-90 |
| 5_36183105 | 5 | 36183105 |  | 97 | 615 | 5,91E-93 | 7,73E-90 |
| 9_37133242 | 9 | 37133242 |  | 95 | 606 | 6,06E-92 | 7,73E-89 |
| 5_36183090 | 5 | 36183090 |  | 88 | 588 | 1,17E-91 | 1,46E-88 |
| 6_152214566 | 6 | 152214566 |  | 1984 | 908 | 5,15E-91 | 6,27E-88 |
| 13_145014100 | 13 | 145014100 | MUC13A | 628 | 1557 | 1,73E-90 | 2,06E-87 |
| 4_139601356 | 4 | 139601356 |  | 826 | 201 | 1,88E-90 | 2,19E-87 |
| 3_99313106 | 3 | 99313106 |  | 1764 | 768 | 2,33E-89 | 2,65E-86 |
| 12_23972389 | 12 | 23972389 |  | 1404 | 537 | 4,73E-89 | 5,27E-86 |
| 2_80538117 | 2 | 80538117 |  | 2217 | 1083 | 2,47E-88 | 2,70E-85 |
| 18_50065548 | 18 | 50065548 | HOXA5 | 217 | 845 | 5,71E-88 | 6,10E-85 |
| 3_125746354 | 3 | 125746354 |  | 214 | 839 | 6,32E-88 | 6,62E-85 |
| 5_60303998 | 5 | 60303998 |  | 219 | 831 | 2,17E-84 | 2,23E-81 |
| 9_119585370 | 9 | 119585370 |  | 161 | 713 | 1,52E-83 | 1,53E-80 |
| 15_86559154 | 15 | 86559154 |  | 446 | 1220 | 4,72E-83 | 4,66E-80 |
| 7_24604695 | 7 | 24604695 | TMP-SLA-2 | 1098 | 375 | 2,30E-82 | 2,23E-79 |
| 6_152214681 | 6 | 152214681 |  | 1870 | 875 | 4,55E-82 | 4,33E-79 |
| 4_139601288 | 4 | 139601288 |  | 813 | 216 | 6,15E-82 | 5,75E-79 |
| 6_44337662 | 6 | 44337662 |  | 657 | 138 | 1,01E-81 | 9,28E-79 |
| 12_43758947 | 12 | 43758947 |  | 1354 | 544 | 2,01E-79 | 1,82E-76 |
| 9_37133142 | 9 | 37133142 |  | 111 | 588 | 2,83E-79 | 2,51E-76 |
| 6_52470400 | 6 | 52470400 |  | 1481 | 629 | 8,67E-79 | 7,57E-76 |
| 12_43758948 | 12 | 43758948 |  | 1348 | 544 | 1,70E-78 | 1,46E-75 |
| 7_24604893 | 7 | 24604893 | TMP-SLA-2 | 1622 | 726 | 4,44E-78 | 3,75E-75 |
| 6_44337644 | 6 | 44337644 |  | 609 | 124 | 1,11E-77 | 9,19E-75 |
| 6_83224589 | 6 | 83224589 |  | 2233 | 1156 | 1,42E-77 | 1,16E-74 |
| 9_37431118 | 9 | 37431118 |  | 66 | 473 | 7,19E-77 | 5,79E-74 |
| 5_36179324 | 5 | 36179324 |  | 103 | 559 | 1,05E-76 | 8,32E-74 |
| 5_36179447 | 5 | 36179447 |  | 73 | 479 | 3,55E-74 | 2,78E-71 |
| 5_36183198 | 5 | 36183198 |  | 41 | 396 | 4,92E-74 | 3,79E-71 |
| 2_107132602 | 2 | 107132602 |  | 318 | 949 | 3,20E-73 | 2,43E-70 |
| 5_97778695 | 5 | 97778695 |  | 269 | 850 | 1,23E-70 | 9,20E-68 |
| 6_62007288 | 6 | 62007288 |  | 4496 | 2966 | 1,37E-70 | 1,01E-67 |
| 7_24604682 | 7 | 24604682 | TMP-SLA-2 | 824 | 257 | 9,83E-70 | 7,15E-67 |
| 13_28951461 | 13 | 28951461 |  | 135 | 595 | 1,08E-69 | 7,74E-67 |
| 4_116885955 | 4 | 116885955 | BCL2L15 | 1065 | 401 | 1,34E-69 | 9,51E-67 |
| 5_42322111 | 5 | 42322111 |  | 340 | 963 | 3,06E-69 | 2,14E-66 |
| 1_82271874 | 1 | 82271874 | CD24 | 140 | 602 | 4,88E-69 | 3,36E-66 |
| 15_55763328 | 15 | 55763328 |  | 131 | 583 | 7,16E-69 | 4,87E-66 |
| 13_145019769 | 13 | 145019769 | MUC13A | 963 | 1883 | 1,14E-67 | 7,63E-65 |
| 6_96416207 | 6 | 96416207 |  | 662 | 1442 | 3,35E-66 | 2,22E-63 |
| 8_12417227 | 8 | 12417227 | ENSSSCG00000028448 | 1076 | 423 | 9,71E-66 | 6,36E-63 |
| 6_152214597 | 6 | 152214597 |  | 1084 | 429 | 1,65E-65 | 1,07E-62 |
| 2_107130936 | 2 | 107130936 |  | 1248 | 537 | 4,19E-65 | 2,67E-62 |
| 8_12417186 | 8 | 12417186 | ENSSSCG00000028448 | 1126 | 459 | 9,58E-65 | 6,04E-62 |
| 17_53826898 | 17 | 53826898 | MMP9 | 553 | 1266 | 3,25E-64 | 2,02E-61 |
| 2_162072451 | 2 | 162072451 |  | 354 | 954 | 6,85E-64 | 4,22E-61 |
| 17_53826925 | 17 | 53826925 | MMP9 | 465 | 1125 | 3,51E-63 | 2,14E-60 |
| 3_99311351 | 3 | 99311351 |  | 44 | 361 | 4,90E-63 | 2,95E-60 |
| 9_3723946 | 9 | 3723946 | ENSSSCG00000014618 | 155 | 593 | 3,74E-61 | 2,23E-58 |
| 2_107132668 | 2 | 107132668 |  | 346 | 920 | 1,89E-60 | 1,11E-57 |
| 5_36185387 | 5 | 36185387 |  | 55 | 378 | 2,34E-60 | 1,36E-57 |
| 5_42322060 | 5 | 42322060 |  | 279 | 808 | 3,77E-60 | 2,17E-57 |
| 15_128593585 | 15 | 128593585 |  | 1309 | 602 | 4,90E-60 | 2,79E-57 |
| 13_145020135 | 13 | 145020135 | MUC13A | 60 | 388 | 7,43E-60 | 4,19E-57 |
| 13_145019310 | 13 | 145019310 | MUC13A | 912 | 1744 | 1,88E-59 | 1,05E-56 |
| 12_41228463 | 12 | 41228463 | CCL5 | 1352 | 635 | 2,45E-59 | 1,35E-56 |
| 14_58841675 | 14 | 58841675 | HEATR1 | 161 | 593 | 6,73E-59 | 3,67E-56 |
| 16_68280836 | 16 | 68280836 |  | 870 | 322 | 1,25E-58 | 6,77E-56 |
| 3_58240175 | 3 | 58240175 |  | 653 | 195 | 1,90E-58 | 1,01E-55 |
| 16_68280840 | 16 | 68280840 |  | 857 | 315 | 2,30E-58 | 1,22E-55 |
| 8_139942865 | 8 | 139942865 | PIGY | 1199 | 539 | 1,15E-57 | 6,02E-55 |
| 16_78288815 | 16 | 78288815 | GPX3 | 567 | 151 | 1,77E-57 | 9,17E-55 |
| 2_75300772 | 2 | 75300772 | EEF2 | 2604 | 1576 | 2,20E-57 | 1,13E-54 |
| 6_152210124 | 6 | 152210124 |  | 851 | 316 | 4,17E-57 | 2,12E-54 |
| 7_36936875 | 7 | 36936875 |  | 56 | 365 | 1,21E-56 | 6,10E-54 |
| 9_73898132 | 9 | 73898132 | PIGR | 93 | 446 | 2,87E-56 | 1,43E-53 |
| 13_82507811 | 13 | 82507811 |  | 166 | 585 | 1,58E-55 | 7,79E-53 |
| 9_73898139 | 9 | 73898139 | PIGR | 96 | 448 | 2,46E-55 | 1,21E-52 |
| 14_92225826 | 14 | 92225826 | C10orf99 | 1155 | 527 | 5,21E-54 | 2,52E-51 |
| 4_122127868 | 4 | 122127868 |  | 275 | 764 | 8,62E-54 | 4,14E-51 |
| 13_144997797 | 13 | 144997797 | MUC13A | 671 | 1360 | 1,24E-53 | 5,90E-51 |
| 6_13128690 | 6 | 13128690 |  | 1518 | 785 | 2,03E-53 | 9,58E-51 |
| 3_60627091 | 3 | 60627091 | CD8B | 126 | 497 | 5,19E-53 | 2,43E-50 |
| 12_23972924 | 12 | 23972924 |  | 868 | 343 | 6,65E-53 | 3,08E-50 |
| 13_76785513 | 13 | 76785513 |  | 725 | 255 | 8,52E-53 | 3,91E-50 |
| 9_146589629 | 9 | 146589629 |  | 870 | 345 | 1,01E-52 | 4,59E-50 |
| 7_134316140 | 7 | 134316140 |  | 121 | 482 | 6,28E-52 | 2,84E-49 |
| 6_152214658 | 6 | 152214658 |  | 885 | 358 | 6,84E-52 | 3,06E-49 |
| 3_99311216 | 3 | 99311216 |  | 93 | 424 | 1,73E-51 | 7,66E-49 |
| 8_139733837 | 8 | 139733837 | ENSSSCG00000026196 | 634 | 206 | 2,11E-51 | 9,27E-49 |
| 6_52593364 | 6 | 52593364 |  | 40 | 303 | 3,81E-51 | 1,65E-48 |
| 14_121828694 | 14 | 121828694 |  | 928 | 389 | 3,81E-51 | 1,66E-48 |
| 2_107132656 | 2 | 107132656 |  | 367 | 894 | 3,96E-51 | 1,70E-48 |
| 16_78290259 | 16 | 78290259 | GPX3 | 576 | 174 | 4,56E-51 | 1,94E-48 |
| 1_308175252 | 1 | 308175252 |  | 83 | 401 | 5,07E-51 | 2,14E-48 |
| 14_14140052 | 14 | 14140052 |  | 818 | 319 | 5,27E-51 | 2,21E-48 |
| 6_83011694 | 6 | 83011694 | RBBP4 | 907 | 378 | 1,57E-50 | 6,54E-48 |
| 6_44973006 | 6 | 44973006 | CYP2B22 | 388 | 922 | 1,77E-50 | 7,28E-48 |
| 3_90545900 | 3 | 90545900 |  | 1629 | 888 | 6,07E-50 | 2,48E-47 |
| 6_44978140 | 6 | 44978140 | CYP2B22 | 438 | 993 | 8,36E-50 | 3,39E-47 |
| 3_99311103 | 3 | 99311103 |  | 106 | 441 | 1,55E-49 | 6,24E-47 |
| 6_44977894 | 6 | 44977894 | CYP2B22 | 313 | 798 | 2,41E-49 | 9,62E-47 |
| 14_149173892 | 14 | 149173892 |  | 42 | 300 | 3,59E-49 | 1,42E-46 |
| 11_4679898 | 11 | 4679898 | POLR1D | 205 | 619 | 4,91E-49 | 1,93E-46 |
| 6_44978094 | 6 | 44978094 | CYP2B22 | 402 | 929 | 2,23E-48 | 8,69E-46 |
| 12_24895670 | 12 | 24895670 |  | 900 | 383 | 2,38E-48 | 9,23E-46 |
| 6_44978095 | 6 | 44978095 | CYP2B22 | 403 | 929 | 3,68E-48 | 1,42E-45 |
| 18_44506477 | 18 | 44506477 |  | 106 | 431 | 1,82E-47 | 6,94E-45 |
| 17_30109282 | 17 | 30109282 | ZNF133 | 413 | 98 | 5,23E-47 | 1,98E-44 |
| 9_37423946 | 9 | 37423946 |  | 214 | 622 | 6,92E-47 | 2,61E-44 |
| 15_55760891 | 15 | 55760891 |  | 310 | 777 | 8,54E-47 | 3,19E-44 |
| 3_99311174 | 3 | 99311174 |  | 85 | 385 | 1,22E-46 | 4,53E-44 |
| 13_156325226 | 13 | 156325226 |  | 599 | 202 | 2,06E-46 | 7,61E-44 |
| 9_3723909 | 9 | 3723909 | ENSSSCG00000014618 | 125 | 462 | 2,23E-46 | 8,18E-44 |
| 6_44977937 | 6 | 44977937 | CYP2B22 | 299 | 757 | 2,26E-46 | 8,20E-44 |
| 6_57852106 | 6 | 57852106 |  | 1182 | 586 | 2,55E-46 | 9,22E-44 |
| 2_107131600 | 2 | 107131600 |  | 1292 | 667 | 6,74E-46 | 2,42E-43 |
| 3_99311196 | 3 | 99311196 |  | 29 | 253 | 8,44E-46 | 3,00E-43 |
| 16_78290583 | 16 | 78290583 | GPX3 | 101 | 412 | 1,43E-45 | 5,07E-43 |
| 13_26045046 | 13 | 26045046 |  | 87 | 383 | 2,43E-45 | 8,54E-43 |
| 4_85390138 | 4 | 85390138 |  | 610 | 213 | 4,15E-45 | 1,45E-42 |
| 3_99311116 | 3 | 99311116 |  | 28 | 246 | 1,06E-44 | 3,66E-42 |
| 6_82337307 | 6 | 82337307 |  | 744 | 299 | 2,40E-44 | 8,25E-42 |
| 9_146589586 | 9 | 146589586 |  | 853 | 372 | 6,40E-44 | 2,19E-41 |
| 14_58845283 | 14 | 58845283 |  | 784 | 327 | 7,88E-44 | 2,68E-41 |
| 6_44970499 | 6 | 44970499 | CYP2B22 | 359 | 831 | 1,43E-43 | 4,82E-41 |
| 5_18667998 | 5 | 18667998 |  | 2154 | 1339 | 1,54E-43 | 5,18E-41 |
| 12_43758879 | 12 | 43758879 |  | 1265 | 666 | 7,64E-43 | 2,55E-40 |
| 2_28657464 | 2 | 28657464 |  | 83 | 363 | 8,18E-43 | 2,71E-40 |
| 12_43759209 | 12 | 43759209 |  | 1334 | 721 | 3,85E-42 | 1,27E-39 |
| 6_44968409 | 6 | 44968409 | CYP2B22 | 472 | 985 | 7,18E-42 | 2,35E-39 |
| 5_42322209 | 5 | 42322209 |  | 299 | 727 | 9,70E-42 | 3,15E-39 |
| X_40398128 | X | 40398128 | ATP6AP2 | 25 | 225 | 2,06E-41 | 6,64E-39 |
| 13_28739886 | 13 | 28739886 | VPAC1 | 53 | 292 | 3,76E-41 | 1,21E-38 |
| 6_152211327 | 6 | 152211327 |  | 469 | 144 | 4,33E-41 | 1,38E-38 |
| 6_63313939 | 6 | 63313939 |  | 890 | 412 | 8,57E-41 | 2,72E-38 |
| 9_39190675 | 9 | 39190675 |  | 949 | 454 | 1,54E-40 | 4,85E-38 |
| 9_119546502 | 9 | 119546502 |  | 255 | 648 | 3,75E-40 | 1,18E-37 |
| 14_43079488 | 14 | 43079488 |  | 446 | 134 | 4,02E-40 | 1,25E-37 |
| 6_53298177 | 6 | 53298177 | RPS9 | 1450 | 822 | 5,19E-40 | 1,61E-37 |
| 9_39190223 | 9 | 39190223 |  | 1116 | 576 | 6,91E-40 | 2,13E-37 |
| 16_6157330 | 16 | 6157330 |  | 139 | 452 | 1,40E-39 | 4,27E-37 |
| 6_152210306 | 6 | 152210306 |  | 780 | 344 | 1,81E-39 | 5,51E-37 |
| 9_144542620 | 9 | 144542620 |  | 99 | 378 | 1,94E-39 | 5,86E-37 |
| 14_92221937 | 14 | 92221937 | C10orf99 | 427 | 126 | 2,99E-39 | 9,01E-37 |
| 9_7436014 | 9 | 7436014 |  | 218 | 583 | 3,31E-39 | 9,90E-37 |
| 12_43758886 | 12 | 43758886 |  | 1314 | 726 | 3,84E-39 | 1,14E-36 |
| 13_213570507 | 13 | 213570507 |  | 61 | 300 | 4,94E-39 | 1,46E-36 |
| 18_41968394 | 18 | 41968394 |  | 595 | 1134 | 6,69E-39 | 1,97E-36 |
| 15_128604656 | 15 | 128604656 |  | 1000 | 501 | 1,67E-38 | 4,89E-36 |
| 3_99311079 | 3 | 99311079 |  | 23 | 208 | 1,87E-38 | 5,44E-36 |
| 13_53672510 | 13 | 53672510 |  | 1257 | 689 | 2,46E-38 | 7,12E-36 |
| 4_15759139 | 4 | 15759139 | TMEM65 | 353 | 89 | 2,90E-38 | 8,33E-36 |
| 5_36179742 | 5 | 36179742 |  | 30 | 225 | 3,99E-38 | 1,14E-35 |
| 2_83955541 | 2 | 83955541 | FCHO2 | 470 | 154 | 4,25E-38 | 1,21E-35 |
| 6_13128490 | 6 | 13128490 |  | 1083 | 563 | 4,40E-38 | 1,25E-35 |
| 14_58842359 | 14 | 58842359 | HEATR1 | 197 | 542 | 4,43E-38 | 1,25E-35 |
| 1_302293956 | 1 | 302293956 |  | 323 | 75 | 9,21E-38 | 2,58E-35 |
| 6_152210319 | 6 | 152210319 |  | 780 | 353 | 1,40E-37 | 3,90E-35 |
| 10_22601895 | 10 | 22601895 |  | 265 | 647 | 1,42E-37 | 3,94E-35 |
| 7_34987416 | 7 | 34987416 | HMGA1 | 765 | 345 | 3,88E-37 | 1,07E-34 |
| 2_93894485 | 2 | 93894485 | VCAN | 518 | 1012 | 4,79E-37 | 1,31E-34 |
| 12_24255307 | 12 | 24255307 |  | 65 | 299 | 5,55E-37 | 1,51E-34 |
| 17_30109288 | 17 | 30109288 | ZNF133 | 430 | 135 | 7,89E-37 | 2,14E-34 |
| 6_13127705 | 6 | 13127705 |  | 616 | 249 | 1,24E-36 | 3,35E-34 |
| 4_107691295 | 4 | 107691295 | CTSS | 751 | 340 | 3,25E-36 | 8,73E-34 |
| 6_44977735 | 6 | 44977735 | CYP2B22 | 380 | 808 | 5,27E-36 | 1,41E-33 |
| 4_116033994 | 4 | 116033994 | CSDE1 | 922 | 1541 | 5,88E-36 | 1,56E-33 |
| 8_71064120 | 8 | 71064120 |  | 381 | 808 | 8,25E-36 | 2,18E-33 |
| 1_91996852 | 1 | 91996852 | NT5DC1 | 239 | 595 | 8,44E-36 | 2,22E-33 |
| 3_49888790 | 3 | 49888790 |  | 419 | 132 | 8,86E-36 | 2,32E-33 |
| 10_16021616 | 10 | 16021616 |  | 616 | 253 | 1,11E-35 | 2,89E-33 |
| 13_142779301 | 13 | 142779301 |  | 727 | 328 | 2,43E-35 | 6,31E-33 |
| 2_82265299 | 2 | 82265299 |  | 211 | 29 | 2,70E-35 | 6,96E-33 |
| 2_107130580 | 2 | 107130580 |  | 1366 | 793 | 3,20E-35 | 8,22E-33 |
| 4_97615788 | 4 | 97615788 |  | 30 | 213 | 3,54E-35 | 9,05E-33 |
| 14_58842003 | 14 | 58842003 | HEATR1 | 155 | 455 | 3,77E-35 | 9,57E-33 |
| 12_23999346 | 12 | 23999346 |  | 117 | 389 | 3,89E-35 | 9,83E-33 |
| 7_37521469 | 7 | 37521469 |  | 1354 | 785 | 4,59E-35 | 1,15E-32 |
| 6_51798085 | 6 | 51798085 | CTU1 | 98 | 354 | 4,66E-35 | 1,17E-32 |
| 14_58842733 | 14 | 58842733 | HEATR1 | 128 | 408 | 4,76E-35 | 1,19E-32 |
| 2_82265302 | 2 | 82265302 |  | 217 | 32 | 5,85E-35 | 1,45E-32 |
| 2_107133112 | 2 | 107133112 |  | 301 | 684 | 6,10E-35 | 1,51E-32 |
| 7_128678589 | 7 | 128678589 |  | 1574 | 956 | 6,37E-35 | 1,57E-32 |
| 13_145019356 | 13 | 145019356 | MUC13A | 157 | 457 | 6,52E-35 | 1,59E-32 |
| 7_128678703 | 7 | 128678703 |  | 1585 | 965 | 7,15E-35 | 1,74E-32 |
| 15_55415891 | 15 | 55415891 | PPAPDC1B | 77 | 313 | 7,51E-35 | 1,82E-32 |
| 16_20345271 | 16 | 20345271 | ENSSSCG00000016817 | 587 | 238 | 8,33E-35 | 2,01E-32 |
| 12_21893564 | 12 | 21893564 |  | 1113 | 606 | 9,67E-35 | 2,32E-32 |
| 14_41263159 | 14 | 41263159 | SLC8B1 | 374 | 110 | 1,11E-34 | 2,66E-32 |
| 2_83955543 | 2 | 83955543 | FCHO2 | 474 | 168 | 1,12E-34 | 2,66E-32 |
| 9_39190667 | 9 | 39190667 |  | 873 | 433 | 1,38E-34 | 3,26E-32 |
| 4_139624733 | 4 | 139624733 |  | 1005 | 529 | 2,22E-34 | 5,23E-32 |
| 13_141230996 | 13 | 141230996 |  | 631 | 269 | 2,97E-34 | 6,98E-32 |
| 16_29452611 | 16 | 29452611 | HMGCS1 | 536 | 208 | 3,31E-34 | 7,74E-32 |
| 6_73042758 | 6 | 73042758 | CDA | 323 | 84 | 3,92E-34 | 9,11E-32 |
| 2_28156702 | 2 | 28156702 |  | 214 | 545 | 3,99E-34 | 9,23E-32 |
| 7_98864040 | 7 | 98864040 |  | 954 | 1563 | 4,04E-34 | 9,32E-32 |
| 2_107132202 | 2 | 107132202 |  | 274 | 637 | 5,08E-34 | 1,17E-31 |
| 16_29452606 | 16 | 29452606 | HMGCS1 | 497 | 185 | 7,24E-34 | 1,66E-31 |
| 13_24849290 | 13 | 24849290 |  | 1036 | 555 | 7,54E-34 | 1,72E-31 |
| 7_5315242 | 7 | 5315242 | BMP6 | 1331 | 778 | 1,20E-33 | 2,71E-31 |
| 7_24643064 | 7 | 24643064 |  | 1068 | 583 | 3,59E-33 | 8,11E-31 |
| 6_44977631 | 6 | 44977631 | CYP2B22 | 278 | 637 | 3,82E-33 | 8,59E-31 |
| 6_73139369 | 6 | 73139369 |  | 1248 | 719 | 4,64E-33 | 1,04E-30 |
| 15_66434767 | 15 | 66434767 | ENSSSCG00000015864 | 1103 | 611 | 7,08E-33 | 1,58E-30 |
| 5_42322183 | 5 | 42322183 |  | 296 | 661 | 9,86E-33 | 2,19E-30 |
| 7_128688144 | 7 | 128688144 |  | 1663 | 1046 | 1,39E-32 | 3,06E-30 |
| 7_128643082 | 7 | 128643082 |  | 430 | 149 | 1,45E-32 | 3,19E-30 |
| 2_107132204 | 2 | 107132204 |  | 281 | 637 | 1,69E-32 | 3,71E-30 |
| 5_106659120 | 5 | 106659120 |  | 53 | 253 | 2,09E-32 | 4,57E-30 |
| 17_12483746 | 17 | 12483746 |  | 197 | 507 | 2,26E-32 | 4,92E-30 |
| 13_37898211 | 13 | 37898211 |  | 586 | 248 | 2,51E-32 | 5,43E-30 |
| 9_37420956 | 9 | 37420956 |  | 46 | 237 | 3,65E-32 | 7,87E-30 |
| 12_21896270 | 12 | 21896270 |  | 1347 | 802 | 3,97E-32 | 8,51E-30 |
| 6_867007 | 6 | 867007 |  | 700 | 326 | 5,03E-32 | 1,07E-29 |
| 14_52259332 | 14 | 52259332 | IGLV-9 | 295 | 75 | 5,83E-32 | 1,24E-29 |
| 17_16443996 | 17 | 16443996 |  | 131 | 396 | 6,02E-32 | 1,28E-29 |
| 5_3970789 | 5 | 3970789 |  | 361 | 111 | 6,59E-32 | 1,39E-29 |
| 7_24749230 | 7 | 24749230 |  | 318 | 88 | 1,20E-31 | 2,52E-29 |
| 5_96182940 | 5 | 96182940 | LUM | 1399 | 847 | 1,51E-31 | 3,16E-29 |
| 2_107133262 | 2 | 107133262 |  | 337 | 711 | 2,33E-31 | 4,87E-29 |
| 10_22598795 | 10 | 22598795 |  | 127 | 385 | 3,29E-31 | 6,81E-29 |
| 6_73139370 | 6 | 73139370 |  | 1230 | 719 | 3,29E-31 | 6,83E-29 |
| X_46764737 | X | 46764737 |  | 366 | 751 | 3,80E-31 | 7,83E-29 |
| 14_149173885 | 14 | 149173885 |  | 56 | 253 | 4,67E-31 | 9,58E-29 |
| 4_122127739 | 4 | 122127739 | SCAMC-1 | 943 | 505 | 5,61E-31 | 1,15E-28 |
| 9_39190161 | 9 | 39190161 |  | 695 | 328 | 5,84E-31 | 1,19E-28 |
| 3_5528127 | 3 | 5528127 |  | 449 | 865 | 7,68E-31 | 1,56E-28 |
| 13_28953802 | 13 | 28953802 |  | 272 | 66 | 7,78E-31 | 1,57E-28 |
| 17_52813966 | 17 | 52813966 | YWHAB | 643 | 293 | 7,77E-31 | 1,57E-28 |
| 15_66434770 | 15 | 66434770 | ENSSSCG00000015864 | 1116 | 636 | 1,09E-30 | 2,19E-28 |
| 7_128678587 | 7 | 128678587 |  | 1527 | 955 | 1,14E-30 | 2,28E-28 |
| 2_162072398 | 2 | 162072398 |  | 268 | 65 | 2,05E-30 | 4,09E-28 |
| 13_156325943 | 13 | 156325943 |  | 1106 | 631 | 2,49E-30 | 4,94E-28 |
| 6_44970168 | 6 | 44970168 | CYP2B22 | 230 | 545 | 2,57E-30 | 5,07E-28 |
| 6_44978486 | 6 | 44978486 | CYP2B22 | 312 | 667 | 2,57E-30 | 5,07E-28 |
| 2_96864478 | 2 | 96864478 | COX7C; | 2892 | 3829 | 2,78E-30 | 5,44E-28 |
| 14_123336129 | 14 | 123336129 |  | 1594 | 1012 | 3,00E-30 | 5,86E-28 |
| 2_6320199 | 2 | 6320199 | BATF2 | 824 | 423 | 3,05E-30 | 5,94E-28 |
| 3_60625257 | 3 | 60625257 | CD8B | 109 | 348 | 3,68E-30 | 7,13E-28 |
| 6_27483135 | 6 | 27483135 |  | 367 | 120 | 3,84E-30 | 7,42E-28 |
| 5_15557405 | 5 | 15557405 |  | 500 | 202 | 4,84E-30 | 9,32E-28 |
| 6_12296791 | 6 | 12296791 |  | 772 | 387 | 4,90E-30 | 9,40E-28 |
| 5_15557330 | 5 | 15557330 |  | 347 | 109 | 5,61E-30 | 1,07E-27 |
| 9_73979923 | 9 | 73979923 | PIGR | 99 | 329 | 6,40E-30 | 1,22E-27 |
| 6_45012267 | 6 | 45012267 |  | 446 | 852 | 8,79E-30 | 1,67E-27 |
| 12_46022955 | 12 | 46022955 |  | 586 | 260 | 1,07E-29 | 2,02E-27 |
| 14_58843050 | 14 | 58843050 | HEATR1 | 91 | 313 | 1,28E-29 | 2,42E-27 |
| 2_136460331 | 2 | 136460331 |  | 421 | 816 | 1,31E-29 | 2,46E-27 |
| 1_280429682 | 1 | 280429682 |  | 298 | 83 | 1,50E-29 | 2,80E-27 |
| 3_80706078 | 3 | 80706078 | RAB1B | 1911 | 1276 | 1,89E-29 | 3,53E-27 |
| 6_44978685 | 6 | 44978685 | CYP2B22 | 244 | 560 | 2,10E-29 | 3,90E-27 |
| 17_15128158 | 17 | 15128158 |  | 33 | 196 | 2,13E-29 | 3,93E-27 |
| 17_53820063 | 17 | 53820063 | MMP9 | 534 | 227 | 2,31E-29 | 4,26E-27 |
| 13_159846575 | 13 | 159846575 | RETNLB | 306 | 88 | 2,54E-29 | 4,66E-27 |
| 12_25326301 | 12 | 25326301 |  | 123 | 367 | 3,13E-29 | 5,73E-27 |
| 2_6320200 | 2 | 6320200 | BATF2 | 814 | 422 | 3,36E-29 | 6,13E-27 |
| 13_76785853 | 13 | 76785853 |  | 516 | 216 | 3,49E-29 | 6,34E-27 |
| 9_3722307 | 9 | 3722307 | ENSSSCG00000014618 | 61 | 254 | 3,84E-29 | 6,95E-27 |
| 16_77975184 | 16 | 77975184 |  | 1321 | 806 | 4,08E-29 | 7,37E-27 |
| 6_87962986 | 6 | 87962986 | NDUFS5 | 388 | 136 | 4,44E-29 | 7,99E-27 |
| 12_21893652 | 12 | 21893652 |  | 394 | 774 | 4,56E-29 | 8,18E-27 |
| 12_22496769 | 12 | 22496769 | THRA | 15 | 149 | 6,27E-29 | 1,12E-26 |
| 9_74206902 | 9 | 74206902 | ENSSSCG00000015663 | 339 | 108 | 7,75E-29 | 1,38E-26 |
| 5_15557276 | 5 | 15557276 |  | 301 | 87 | 1,03E-28 | 1,83E-26 |
| 9_74206901 | 9 | 74206901 | ENSSSCG00000015663 | 338 | 108 | 1,18E-28 | 2,08E-26 |
| 9_133634809 | 9 | 133634809 | QSOX1 | 164 | 431 | 1,26E-28 | 2,22E-26 |
| 12_21896288 | 12 | 21896288 |  | 1507 | 958 | 1,51E-28 | 2,64E-26 |
| 14_140504897 | 14 | 140504897 |  | 671 | 324 | 1,51E-28 | 2,65E-26 |
| 16_6152668 | 16 | 6152668 |  | 354 | 118 | 1,94E-28 | 3,38E-26 |
| 7_25332107 | 7 | 25332107 | UBD | 1621 | 1054 | 4,53E-28 | 7,88E-26 |
| 9_73979926 | 9 | 73979926 | PIGR | 98 | 317 | 5,13E-28 | 8,89E-26 |
| 16_29451988 | 16 | 29451988 | HMGCS1 | 1357 | 846 | 9,78E-28 | 1,69E-25 |
| 7_27590174 | 7 | 27590174 | BAT1 | 376 | 738 | 9,93E-28 | 1,71E-25 |
| 2_60643361 | 2 | 60643361 | SLC35E1 | 297 | 88 | 1,32E-27 | 2,26E-25 |
| 3_99313453 | 3 | 99313453 |  | 286 | 82 | 1,39E-27 | 2,38E-25 |
| 8_80738049 | 8 | 80738049 |  | 565 | 256 | 1,42E-27 | 2,42E-25 |
| 2_83190670 | 2 | 83190670 | KIAA1191 | 1352 | 844 | 1,64E-27 | 2,79E-25 |
| 13_213574231 | 13 | 213574231 |  | 357 | 123 | 1,75E-27 | 2,96E-25 |
| 11_2161405 | 11 | 2161405 |  | 398 | 148 | 1,90E-27 | 3,20E-25 |
| 5_7489906 | 5 | 7489906 |  | 688 | 342 | 1,92E-27 | 3,22E-25 |
| 1_280429687 | 1 | 280429687 |  | 294 | 87 | 2,22E-27 | 3,73E-25 |
| 17_53824674 | 17 | 53824674 | MMP9 | 402 | 771 | 2,34E-27 | 3,91E-25 |
| 14_140603890 | 14 | 140603890 |  | 1359 | 851 | 2,41E-27 | 4,02E-25 |
| 18_44506684 | 18 | 44506684 |  | 92 | 302 | 3,33E-27 | 5,54E-25 |
| 12_21894165 | 12 | 21894165 |  | 98 | 312 | 4,26E-27 | 7,07E-25 |
| 6_43734003 | 6 | 43734003 |  | 688 | 344 | 4,34E-27 | 7,17E-25 |
| 14_14140016 | 14 | 14140016 |  | 1002 | 576 | 5,06E-27 | 8,32E-25 |
| 2_107131615 | 2 | 107131615 |  | 1304 | 811 | 6,12E-27 | 1,01E-24 |
| 16_68279841 | 16 | 68279841 |  | 612 | 292 | 7,84E-27 | 1,28E-24 |
| 14_98578470 | 14 | 98578470 |  | 1231 | 755 | 9,12E-27 | 1,49E-24 |
| 2_107133227 | 2 | 107133227 |  | 371 | 723 | 9,56E-27 | 1,55E-24 |
| 2_8584274 | 2 | 8584274 |  | 244 | 62 | 1,11E-26 | 1,79E-24 |
| 17_36244460 | 17 | 36244460 |  | 517 | 228 | 1,14E-26 | 1,85E-24 |
| 7_24749232 | 7 | 24749232 |  | 306 | 96 | 1,22E-26 | 1,97E-24 |
| 7_29113048 | 7 | 29113048 |  | 518 | 229 | 1,34E-26 | 2,16E-24 |
| 14_149178114 | 14 | 149178114 |  | 65 | 249 | 1,56E-26 | 2,51E-24 |
| 6_152210279 | 6 | 152210279 |  | 284 | 598 | 1,67E-26 | 2,66E-24 |
| 9_148559237 | 9 | 148559237 |  | 1476 | 953 | 2,10E-26 | 3,35E-24 |
| 9_119585586 | 9 | 119585586 |  | 108 | 325 | 2,52E-26 | 4,00E-24 |
| 9_74206952 | 9 | 74206952 | ENSSSCG00000015663 | 370 | 135 | 2,58E-26 | 4,07E-24 |
| 12_43758774 | 12 | 43758774 |  | 1340 | 845 | 2,57E-26 | 4,07E-24 |
| 9_21861398 | 9 | 21861398 |  | 285 | 598 | 2,59E-26 | 4,07E-24 |
| 2_84006275 | 2 | 84006275 | TMEM171 | 1137 | 686 | 3,19E-26 | 5,00E-24 |
| 17_46855475 | 17 | 46855475 | LBP | 47 | 212 | 3,33E-26 | 5,20E-24 |
| 12_25324032 | 12 | 25324032 |  | 38 | 193 | 3,60E-26 | 5,61E-24 |
| 5_7489900 | 5 | 7489900 |  | 700 | 358 | 3,81E-26 | 5,91E-24 |
| 6_152210822 | 6 | 152210822 |  | 425 | 792 | 4,00E-26 | 6,19E-24 |
| 6_63313955 | 6 | 63313955 |  | 920 | 521 | 4,95E-26 | 7,64E-24 |
| 12_26664520 | 12 | 26664520 | ENSSSCG00000028962 | 59 | 235 | 4,99E-26 | 7,69E-24 |
| 12_43758990 | 12 | 43758990 |  | 1261 | 785 | 5,14E-26 | 7,89E-24 |
| 12_25321309 | 12 | 25321309 |  | 37 | 190 | 5,57E-26 | 8,52E-24 |
| 10_16021588 | 10 | 16021588 |  | 632 | 311 | 6,69E-26 | 1,02E-23 |
| 13_141227225 | 13 | 141227225 |  | 663 | 333 | 6,80E-26 | 1,03E-23 |
| 1_280430244 | 1 | 280430244 |  | 236 | 60 | 7,73E-26 | 1,17E-23 |
| 17_34560290 | 17 | 34560290 |  | 1228 | 1806 | 8,02E-26 | 1,21E-23 |
| 5_9322735 | 5 | 9322735 | ENSSSCG00000020882 | 25 | 162 | 9,04E-26 | 1,36E-23 |
| 2_5942665 | 2 | 5942665 |  | 1557 | 1025 | 9,91E-26 | 1,49E-23 |
| 6_16253802 | 6 | 16253802 |  | 64 | 243 | 1,04E-25 | 1,56E-23 |
| 3_99311554 | 3 | 99311554 |  | 26 | 164 | 1,10E-25 | 1,65E-23 |
| 5_23172774 | 5 | 23172774 |  | 567 | 267 | 1,20E-25 | 1,79E-23 |
| 12_5257698 | 12 | 5257698 | EXOC7 | 634 | 314 | 1,30E-25 | 1,92E-23 |
| 4_101227694 | 4 | 101227694 |  | 517 | 233 | 1,29E-25 | 1,92E-23 |
| 6_70509778 | 6 | 70509778 |  | 184 | 443 | 1,36E-25 | 2,01E-23 |
| 14_110189134 | 14 | 110189134 | IFIT2 | 36 | 186 | 1,44E-25 | 2,13E-23 |
| 7_128678478 | 7 | 128678478 |  | 1487 | 970 | 1,49E-25 | 2,19E-23 |
| 17_46907177 | 17 | 46907177 |  | 1617 | 1076 | 1,61E-25 | 2,37E-23 |
| 12_43758772 | 12 | 43758772 |  | 1331 | 845 | 1,62E-25 | 2,37E-23 |
| 17_52813990 | 17 | 52813990 | YWHAB | 444 | 185 | 1,64E-25 | 2,39E-23 |
| 4_20267760 | 4 | 20267760 | TAF2 | 130 | 357 | 1,65E-25 | 2,39E-23 |
| 4_116887338 | 4 | 116887338 | BCL2L15 | 17 | 141 | 1,70E-25 | 2,47E-23 |
| 11_27051932 | 11 | 27051932 | OLFM4 | 278 | 84 | 2,22E-25 | 3,21E-23 |
| 18_55732111 | 18 | 55732111 |  | 642 | 321 | 2,24E-25 | 3,23E-23 |
| 10_22597885 | 10 | 22597885 |  | 109 | 321 | 2,54E-25 | 3,66E-23 |
| 6_17957536 | 6 | 17957536 | MMP15 | 180 | 435 | 2,58E-25 | 3,70E-23 |
| 17_53184077 | 17 | 53184077 |  | 1011 | 1534 | 2,94E-25 | 4,20E-23 |
| 6_83011536 | 6 | 83011536 | RBBP4 | 212 | 483 | 3,16E-25 | 4,51E-23 |
| 6_51798079 | 6 | 51798079 | CTU1 | 103 | 310 | 3,54E-25 | 5,03E-23 |
| 12_23441129 | 12 | 23441129 |  | 65 | 242 | 3,92E-25 | 5,56E-23 |
| 1_302804565 | 1 | 302804565 | URM1 | 400 | 159 | 5,92E-25 | 8,38E-23 |
| 15_54770678 | 15 | 54770678 |  | 82 | 272 | 6,35E-25 | 8,96E-23 |
| 6_13127674 | 6 | 13127674 |  | 641 | 323 | 6,71E-25 | 9,44E-23 |
| X_97963683 | X | 97963683 |  | 25 | 158 | 8,14E-25 | 1,14E-22 |
| 7_1783395 | 7 | 1783395 |  | 191 | 40 | 8,68E-25 | 1,22E-22 |
| 18_7717027 | 18 | 7717027 |  | 115 | 327 | 1,28E-24 | 1,79E-22 |
| 5_17309854 | 5 | 17309854 | DAZAP2 | 157 | 25 | 1,41E-24 | 1,96E-22 |
| 2_28656211 | 2 | 28656211 |  | 1352 | 1938 | 1,52E-24 | 2,11E-22 |
| 16_78282373 | 16 | 78282373 | GPX3 | 485 | 858 | 1,66E-24 | 2,30E-22 |
| 4_139601039 | 4 | 139601039 | GBP4 | 859 | 486 | 1,80E-24 | 2,49E-22 |
| 2_61922695 | 2 | 61922695 | BRD4 | 37 | 183 | 2,03E-24 | 2,80E-22 |
| 10_47234866 | 10 | 47234866 |  | 709 | 375 | 2,07E-24 | 2,85E-22 |
| 9_146592055 | 9 | 146592055 |  | 582 | 284 | 2,09E-24 | 2,87E-22 |
| 6_152708763 | 6 | 152708763 |  | 589 | 289 | 2,16E-24 | 2,95E-22 |
| 2_83177045 | 2 | 83177045 | KIAA1191 | 887 | 508 | 2,35E-24 | 3,20E-22 |
| 2_83139632 | 2 | 83139632 |  | 3763 | 2930 | 2,37E-24 | 3,22E-22 |
| 3_51609880 | 3 | 51609880 |  | 819 | 457 | 2,57E-24 | 3,48E-22 |
| 17_52283934 | 17 | 52283934 | HNF4A | 765 | 417 | 2,78E-24 | 3,75E-22 |
| 6_44978380 | 6 | 44978380 | CYP2B22 | 328 | 643 | 2,78E-24 | 3,76E-22 |
| 6_152211909 | 6 | 152211909 |  | 313 | 108 | 3,20E-24 | 4,30E-22 |
| 3_58218159 | 3 | 58218159 |  | 155 | 389 | 3,25E-24 | 4,36E-22 |
| 5_36185519 | 5 | 36185519 |  | 28 | 162 | 3,97E-24 | 5,31E-22 |
| 8_124186903 | 8 | 124186903 |  | 43 | 194 | 4,30E-24 | 5,74E-22 |
| 9_74205765 | 9 | 74205765 | ENSSSCG00000015663 | 297 | 99 | 4,40E-24 | 5,86E-22 |
| 6_12296958 | 6 | 12296958 |  | 811 | 453 | 5,02E-24 | 6,66E-22 |
| 4_139601980 | 4 | 139601980 |  | 37 | 181 | 5,63E-24 | 7,47E-22 |
| 15_133426023 | 15 | 133426023 |  | 561 | 273 | 1,02E-23 | 1,35E-21 |
| 8_140656446 | 8 | 140656446 |  | 563 | 951 | 1,51E-23 | 1,99E-21 |
| 2_12862929 | 2 | 12862929 |  | 394 | 161 | 1,53E-23 | 2,01E-21 |
| 6_70504664 | 6 | 70504664 |  | 334 | 645 | 1,64E-23 | 2,15E-21 |
| 18_55403002 | 18 | 55403002 |  | 410 | 172 | 2,12E-23 | 2,78E-21 |
| 9_67492388 | 9 | 67492388 | ENSSSCG00000025707 | 277 | 90 | 2,88E-23 | 3,76E-21 |
| 5_3358174 | 5 | 3358174 | PACSIN2 | 392 | 161 | 3,09E-23 | 4,02E-21 |
| 3_48092760 | 3 | 48092760 |  | 113 | 10 | 3,09E-23 | 4,02E-21 |
| 13_24854695 | 13 | 24854695 |  | 882 | 513 | 3,73E-23 | 4,84E-21 |
| 6_152708760 | 6 | 152708760 |  | 583 | 292 | 4,32E-23 | 5,58E-21 |
| 13_145019855 | 13 | 145019855 | MUC13A | 190 | 435 | 4,60E-23 | 5,93E-21 |
| 3_24701485 | 3 | 24701485 |  | 1526 | 1027 | 4,72E-23 | 6,07E-21 |
| 2_28656208 | 2 | 28656208 |  | 1386 | 1957 | 4,97E-23 | 6,38E-21 |
| 2_107130628 | 2 | 107130628 |  | 1152 | 725 | 5,32E-23 | 6,81E-21 |
| 12_19898871 | 12 | 19898871 |  | 84 | 265 | 5,62E-23 | 7,17E-21 |
| 6_69343949 | 6 | 69343949 |  | 247 | 518 | 5,79E-23 | 7,38E-21 |
| 6_70517629 | 6 | 70517629 |  | 188 | 431 | 6,41E-23 | 8,15E-21 |
| 1_283975827 | 1 | 283975827 | HSDL2 | 52 | 206 | 7,24E-23 | 9,18E-21 |
| 12_61907454 | 12 | 61907454 | ENSSSCG00000018027 | 828 | 474 | 7,29E-23 | 9,21E-21 |
| 7_102788208 | 7 | 102788208 |  | 76 | 250 | 8,32E-23 | 1,05E-20 |
| 4_142110237 | 4 | 142110237 |  | 276 | 91 | 8,83E-23 | 1,11E-20 |
| 13_24849358 | 13 | 24849358 |  | 1090 | 678 | 9,32E-23 | 1,17E-20 |
| 9_74205801 | 9 | 74205801 | ENSSSCG00000015663 | 260 | 82 | 1,06E-22 | 1,33E-20 |
| 9_146589538 | 9 | 146589538 |  | 748 | 415 | 1,10E-22 | 1,37E-20 |
| 10_38214362 | 10 | 38214362 |  | 1537 | 1040 | 1,12E-22 | 1,39E-20 |
| 6_119272860 | 6 | 119272860 | MCOLN2 | 31 | 162 | 1,33E-22 | 1,65E-20 |
| 13_34420616 | 13 | 34420616 | TMA7 | 58 | 216 | 1,40E-22 | 1,74E-20 |
| 4_98093261 | 4 | 98093261 | PEX19 | 875 | 512 | 1,43E-22 | 1,77E-20 |
| 7_7781312 | 7 | 7781312 | ENSSSCG00000001038 | 2140 | 1547 | 1,51E-22 | 1,86E-20 |
| 9_3724253 | 9 | 3724253 | ENSSSCG00000014618 | 15 | 124 | 1,59E-22 | 1,96E-20 |
| 12_37434217 | 12 | 37434217 | RPS6KB1 | 174 | 407 | 1,68E-22 | 2,07E-20 |
| 16_78290162 | 16 | 78290162 | GPX3 | 68 | 234 | 1,70E-22 | 2,09E-20 |
| 9_3721954 | 9 | 3721954 | ENSSSCG00000014618 | 29 | 157 | 1,84E-22 | 2,25E-20 |
| 7_42088309 | 7 | 42088309 | FOXP4 | 25 | 148 | 1,87E-22 | 2,27E-20 |
| 18_6549806 | 18 | 6549806 |  | 241 | 72 | 1,86E-22 | 2,27E-20 |
| 9_3721967 | 9 | 3721967 | ENSSSCG00000014618 | 30 | 159 | 2,04E-22 | 2,48E-20 |
| 9_74207689 | 9 | 74207689 | ENSSSCG00000015663 | 385 | 160 | 2,08E-22 | 2,53E-20 |
| 13_156326976 | 13 | 156326976 |  | 538 | 264 | 2,11E-22 | 2,56E-20 |
| 7_60249084 | 7 | 60249084 |  | 114 | 312 | 2,30E-22 | 2,78E-20 |
| 9_67492360 | 9 | 67492360 | ENSSSCG00000025707 | 287 | 99 | 2,48E-22 | 2,99E-20 |
| 7_26684622 | 7 | 26684622 |  | 954 | 575 | 2,56E-22 | 3,07E-20 |
| 7_44082237 | 7 | 44082237 | ENSSSCG00000001688 | 38 | 175 | 3,31E-22 | 3,97E-20 |
| 17_52283931 | 17 | 52283931 | HNF4A | 751 | 421 | 3,87E-22 | 4,63E-20 |
| 14_145335409 | 14 | 145335409 | OAT | 686 | 373 | 4,53E-22 | 5,40E-20 |
| 16_29453169 | 16 | 29453169 | HMGCS1 | 376 | 690 | 4,59E-22 | 5,47E-20 |
| 14_58842625 | 14 | 58842625 | HEATR1 | 155 | 375 | 4,65E-22 | 5,52E-20 |
| 9_3723256 | 9 | 3723256 | ENSSSCG00000014618 | 12 | 114 | 5,10E-22 | 6,05E-20 |
| 4_102857627 | 4 | 102857627 | KIAA0907 | 227 | 482 | 5,16E-22 | 6,10E-20 |
| 14_58843809 | 14 | 58843809 |  | 98 | 283 | 6,27E-22 | 7,39E-20 |
| 16_6152686 | 16 | 6152686 |  | 311 | 115 | 6,29E-22 | 7,40E-20 |
| 2_9703169 | 2 | 9703169 |  | 729 | 406 | 6,39E-22 | 7,50E-20 |
| 16_68279348 | 16 | 68279348 |  | 20 | 134 | 6,54E-22 | 7,66E-20 |
| 7_34162029 | 7 | 34162029 |  | 16 | 124 | 7,02E-22 | 8,20E-20 |
| 8_139942779 | 8 | 139942779 | PIGY | 1308 | 861 | 7,23E-22 | 8,43E-20 |
| 9_3991913 | 9 | 3991913 |  | 216 | 60 | 7,59E-22 | 8,83E-20 |
| 17_34561368 | 17 | 34561368 |  | 29 | 154 | 8,88E-22 | 1,03E-19 |
| 8_10839083 | 8 | 10839083 |  | 15 | 121 | 9,04E-22 | 1,05E-19 |
| 11_27051941 | 11 | 27051941 | OLFM4 | 258 | 84 | 1,04E-21 | 1,20E-19 |
| 13_145020553 | 13 | 145020553 | MUC13A | 531 | 891 | 1,06E-21 | 1,22E-19 |
| 13_213572210 | 13 | 213572210 |  | 352 | 654 | 1,15E-21 | 1,32E-19 |
| 2_162096852 | 2 | 162096852 | ENSSSCG00000014565 | 27 | 149 | 1,21E-21 | 1,39E-19 |
| 5_65447509 | 5 | 65447509 |  | 28 | 151 | 1,35E-21 | 1,55E-19 |
| 13_24854773 | 13 | 24854773 |  | 714 | 397 | 1,35E-21 | 1,55E-19 |
| 14_58842809 | 14 | 58842809 | HEATR1 | 95 | 276 | 1,38E-21 | 1,57E-19 |
| 14_9498534 | 14 | 9498534 | ADAMDEC1 | 285 | 561 | 1,42E-21 | 1,62E-19 |
| 6_83227798 | 6 | 83227798 |  | 1834 | 2459 | 1,42E-21 | 1,62E-19 |
| 15_133426001 | 15 | 133426001 |  | 672 | 366 | 1,48E-21 | 1,68E-19 |
| 3_99311570 | 3 | 99311570 |  | 29 | 153 | 1,50E-21 | 1,69E-19 |
| 4_70412609 | 4 | 70412609 | TRAM1 | 25 | 144 | 1,60E-21 | 1,80E-19 |
| 14_58844459 | 14 | 58844459 |  | 920 | 555 | 1,63E-21 | 1,83E-19 |
| 12_20283016 | 12 | 20283016 |  | 80 | 250 | 1,63E-21 | 1,83E-19 |
| 7_128678382 | 7 | 128678382 |  | 798 | 461 | 1,63E-21 | 1,83E-19 |
| 4_38942721 | 4 | 38942721 |  | 1954 | 1403 | 1,84E-21 | 2,06E-19 |
| X_143687932 | X | 143687932 |  | 20 | 132 | 1,98E-21 | 2,21E-19 |
| 14_58842769 | 14 | 58842769 | HEATR1 | 104 | 290 | 2,01E-21 | 2,24E-19 |
| 17_36283855 | 17 | 36283855 |  | 479 | 228 | 2,02E-21 | 2,25E-19 |
| 13_145010257 | 13 | 145010257 | MUC13A | 529 | 885 | 2,29E-21 | 2,54E-19 |
| 2_77790641 | 2 | 77790641 |  | 275 | 545 | 2,53E-21 | 2,80E-19 |
| 6_43752015 | 6 | 43752015 |  | 55 | 204 | 2,57E-21 | 2,84E-19 |
| 11_4680580 | 11 | 4680580 |  | 1725 | 1212 | 2,76E-21 | 3,04E-19 |
| 13_144183088 | 13 | 144183088 |  | 536 | 269 | 2,99E-21 | 3,29E-19 |
| 7_24686061 | 7 | 24686061 |  | 52 | 198 | 3,02E-21 | 3,31E-19 |
| 6_957389 | 6 | 957389 |  | 351 | 143 | 3,16E-21 | 3,46E-19 |
| 15_133507323 | 15 | 133507323 | VIL1 | 1462 | 994 | 3,33E-21 | 3,64E-19 |
| 6_88650456 | 6 | 88650456 | ENSSSCG00000003664 | 580 | 301 | 3,53E-21 | 3,85E-19 |
| 9_7548456 | 9 | 7548456 | FOLR2 | 110 | 298 | 4,04E-21 | 4,39E-19 |
| 18_7717011 | 18 | 7717011 |  | 94 | 271 | 5,18E-21 | 5,63E-19 |
| 14_14140239 | 14 | 14140239 |  | 520 | 259 | 5,22E-21 | 5,66E-19 |
| 6_52532097 | 6 | 52532097 |  | 1218 | 1728 | 5,40E-21 | 5,84E-19 |
| 7_27852053 | 7 | 27852053 | C7H6ORF29 | 377 | 161 | 5,51E-21 | 5,95E-19 |
| 7_35230222 | 7 | 35230222 | SPDEF | 564 | 291 | 6,43E-21 | 6,93E-19 |
| 5_38418717 | 5 | 38418717 | TMEM19 | 355 | 147 | 6,79E-21 | 7,30E-19 |
| 12_62606579 | 12 | 62606579 |  | 220 | 65 | 7,34E-21 | 7,88E-19 |
| 4_122128098 | 4 | 122128098 |  | 740 | 1146 | 7,84E-21 | 8,39E-19 |
| 18_53426750 | 18 | 53426750 |  | 1086 | 692 | 8,09E-21 | 8,65E-19 |
| 7_21022796 | 7 | 21022796 |  | 130 | 328 | 8,30E-21 | 8,85E-19 |
| 9_3724478 | 9 | 3724478 | ENSSSCG00000014618 | 283 | 102 | 8,48E-21 | 9,03E-19 |
| 13_141231577 | 13 | 141231577 |  | 564 | 292 | 9,46E-21 | 1,00E-18 |
| 2_9703044 | 2 | 9703044 |  | 735 | 419 | 1,05E-20 | 1,11E-18 |
| 4_139591914 | 4 | 139591914 | GBP4 | 485 | 236 | 1,06E-20 | 1,12E-18 |
| 8_31537614 | 8 | 31537614 | KLF3 | 475 | 229 | 1,06E-20 | 1,12E-18 |
| 5_6786725 | 5 | 6786725 |  | 679 | 377 | 1,09E-20 | 1,15E-18 |
| 7_27852058 | 7 | 27852058 | C7H6ORF29 | 370 | 158 | 1,27E-20 | 1,33E-18 |
| 9_67490086 | 9 | 67490086 | ENSSSCG00000025707 | 272 | 96 | 1,30E-20 | 1,37E-18 |
| 6_13128607 | 6 | 13128607 |  | 1199 | 785 | 1,33E-20 | 1,39E-18 |
| 1_302294950 | 1 | 302294950 |  | 842 | 502 | 1,45E-20 | 1,52E-18 |
| 3_99313668 | 3 | 99313668 |  | 461 | 220 | 1,47E-20 | 1,53E-18 |
| 17_66513527 | 17 | 66513527 |  | 1711 | 1209 | 1,48E-20 | 1,54E-18 |
| 2_121515655 | 2 | 121515655 |  | 695 | 390 | 1,54E-20 | 1,60E-18 |
| 7_28044031 | 7 | 28044031 |  | 1213 | 797 | 1,55E-20 | 1,61E-18 |
| 2_134987790 | 2 | 134987790 |  | 845 | 505 | 1,77E-20 | 1,83E-18 |
| 2_66506718 | 2 | 66506718 | JUNB | 1569 | 1091 | 1,80E-20 | 1,86E-18 |
| 2_141885979 | 2 | 141885979 |  | 1672 | 1178 | 2,09E-20 | 2,15E-18 |
| 6_44977908 | 6 | 44977908 | CYP2B22 | 140 | 341 | 2,09E-20 | 2,15E-18 |
| 5_86806302 | 5 | 86806302 | ARL1 | 598 | 319 | 2,20E-20 | 2,26E-18 |
| 4_139591929 | 4 | 139591929 | GBP4 | 529 | 269 | 2,22E-20 | 2,27E-18 |
| 6_75858398 | 6 | 75858398 |  | 235 | 75 | 2,30E-20 | 2,34E-18 |
| 10_64845928 | 10 | 64845928 |  | 31 | 152 | 2,29E-20 | 2,34E-18 |
| 7_128696288 | 7 | 128696288 |  | 1383 | 938 | 2,37E-20 | 2,41E-18 |
| 12_37337333 | 12 | 37337333 | VMP1 | 174 | 392 | 2,51E-20 | 2,56E-18 |
| 7_35230227 | 7 | 35230227 | SPDEF | 563 | 294 | 2,68E-20 | 2,72E-18 |
| 12_43759021 | 12 | 43759021 |  | 973 | 607 | 2,87E-20 | 2,91E-18 |
| 18_52037947 | 18 | 52037947 | CYCS; | 835 | 499 | 2,95E-20 | 2,98E-18 |
| 13_74357623 | 13 | 74357623 |  | 131 | 326 | 2,99E-20 | 3,01E-18 |
| 6_13128943 | 6 | 13128943 |  | 1042 | 662 | 2,99E-20 | 3,01E-18 |
| 14_9503391 | 14 | 9503391 | ADAMDEC1 | 819 | 1236 | 3,32E-20 | 3,33E-18 |
| 17_52295669 | 17 | 52295669 | HNF4A | 735 | 423 | 3,75E-20 | 3,76E-18 |
| 17_34561339 | 17 | 34561339 |  | 25 | 138 | 3,89E-20 | 3,89E-18 |
| X_122175621 | X | 122175621 |  | 5 | 86 | 3,98E-20 | 3,98E-18 |
| 5_36181011 | 5 | 36181011 |  | 10 | 101 | 4,40E-20 | 4,38E-18 |
| 12_24903597 | 12 | 24903597 |  | 1002 | 632 | 4,84E-20 | 4,82E-18 |
| 9_74207774 | 9 | 74207774 | ENSSSCG00000015663 | 343 | 143 | 5,21E-20 | 5,17E-18 |
| 2_5810657 | 2 | 5810657 |  | 1055 | 675 | 5,76E-20 | 5,71E-18 |
| 9_39190618 | 9 | 39190618 |  | 1293 | 869 | 7,08E-20 | 7,01E-18 |
| 10_64845970 | 10 | 64845970 |  | 41 | 170 | 7,33E-20 | 7,24E-18 |
| 12_21539696 | 12 | 21539696 | KRT19 | 1050 | 672 | 7,41E-20 | 7,31E-18 |
| 13_53672669 | 13 | 53672669 |  | 1474 | 1019 | 7,66E-20 | 7,54E-18 |
| 2_84006263 | 2 | 84006263 | TMEM171 | 1325 | 896 | 8,25E-20 | 8,10E-18 |
| 6_70509709 | 6 | 70509709 |  | 199 | 425 | 8,36E-20 | 8,19E-18 |
| 6_70509715 | 6 | 70509715 |  | 213 | 445 | 8,95E-20 | 8,76E-18 |
| 9_67490087 | 9 | 67490087 | ENSSSCG00000025707 | 267 | 96 | 9,19E-20 | 8,98E-18 |
| 2_53871624 | 2 | 53871624 | C1orf35 | 781 | 1183 | 1,09E-19 | 1,06E-17 |
| 6_52448998 | 6 | 52448998 |  | 313 | 125 | 1,10E-19 | 1,07E-17 |
| 8_145146437 | 8 | 145146437 |  | 374 | 165 | 1,13E-19 | 1,10E-17 |
| 15_87899810 | 15 | 87899810 | ITGA6 | 14 | 110 | 1,17E-19 | 1,13E-17 |
| 13_131634934 | 13 | 131634934 | PSMD2 | 63 | 210 | 1,20E-19 | 1,16E-17 |
| 11_71561180 | 11 | 71561180 |  | 40 | 167 | 1,20E-19 | 1,16E-17 |
| 18_6509885 | 18 | 6509885 | FASTK | 444 | 213 | 1,21E-19 | 1,17E-17 |
| 4_85390196 | 4 | 85390196 |  | 636 | 352 | 1,23E-19 | 1,19E-17 |
| 2_5942663 | 2 | 5942663 |  | 1478 | 1025 | 1,32E-19 | 1,26E-17 |
| 14_58843618 | 14 | 58843618 |  | 137 | 331 | 1,37E-19 | 1,31E-17 |
| 5_42322223 | 5 | 42322223 |  | 90 | 256 | 1,41E-19 | 1,35E-17 |
| 13_21242656 | 13 | 21242656 |  | 80 | 239 | 1,53E-19 | 1,46E-17 |
| 13_33067417 | 13 | 33067417 |  | 277 | 103 | 1,59E-19 | 1,51E-17 |
| 5_18900522 | 5 | 18900522 | SPRYD3 | 237 | 79 | 1,72E-19 | 1,64E-17 |
| 7_48120566 | 7 | 48120566 | ENSSSCG00000001720 | 242 | 82 | 1,79E-19 | 1,70E-17 |
| 10_47233408 | 10 | 47233408 |  | 710 | 409 | 1,84E-19 | 1,74E-17 |
| 4_134020790 | 4 | 134020790 |  | 416 | 719 | 1,92E-19 | 1,82E-17 |
| 1_280430252 | 1 | 280430252 |  | 228 | 74 | 2,06E-19 | 1,95E-17 |
| 2_107146641 | 2 | 107146641 |  | 289 | 111 | 2,18E-19 | 2,06E-17 |
| 13_24867845 | 13 | 24867845 |  | 700 | 402 | 2,22E-19 | 2,09E-17 |
| 4_110270838 | 4 | 110270838 |  | 243 | 83 | 2,35E-19 | 2,21E-17 |
| 2_134986875 | 2 | 134986875 | ENSSSCG00000014247 | 615 | 339 | 3,07E-19 | 2,88E-17 |
| 17_36196678 | 17 | 36196678 |  | 252 | 89 | 3,53E-19 | 3,31E-17 |
| 7_94582307 | 7 | 94582307 |  | 724 | 422 | 3,78E-19 | 3,53E-17 |
| 1_3733018 | 1 | 3733018 | SFT2D1 | 423 | 201 | 3,79E-19 | 3,54E-17 |
| 13_149711945 | 13 | 149711945 |  | 666 | 378 | 3,92E-19 | 3,65E-17 |
| 12_24901587 | 12 | 24901587 |  | 549 | 291 | 3,96E-19 | 3,68E-17 |
| 14_11708652 | 14 | 11708652 | DPYSL2 | 54 | 191 | 3,96E-19 | 3,68E-17 |
| 14_81787121 | 14 | 81787121 |  | 331 | 139 | 3,98E-19 | 3,68E-17 |
| 14_149175213 | 14 | 149175213 |  | 46 | 176 | 4,11E-19 | 3,80E-17 |
| 6_155491947 | 6 | 155491947 | EBNA1BP2 | 836 | 509 | 4,15E-19 | 3,83E-17 |
| 2_9703058 | 2 | 9703058 |  | 700 | 404 | 4,18E-19 | 3,86E-17 |
| 9_74207709 | 9 | 74207709 | ENSSSCG00000015663 | 365 | 162 | 5,03E-19 | 4,62E-17 |
| 5_23675286 | 5 | 23675286 |  | 645 | 363 | 5,15E-19 | 4,73E-17 |
| 14_149178431 | 14 | 149178431 |  | 229 | 76 | 5,61E-19 | 5,14E-17 |
| 6_52794778 | 6 | 52794778 | ENSSSCG00000028154 | 267 | 99 | 6,04E-19 | 5,53E-17 |
| 6_49004628 | 6 | 49004628 | SEPW1 | 137 | 27 | 6,39E-19 | 5,84E-17 |
| 12_46033660 | 12 | 46033660 |  | 414 | 196 | 6,57E-19 | 5,99E-17 |
| 12_24901250 | 12 | 24901250 |  | 955 | 605 | 7,11E-19 | 6,47E-17 |
| 2_134987341 | 2 | 134987341 |  | 555 | 297 | 7,13E-19 | 6,48E-17 |
| 12_19216234 | 12 | 19216234 | GRN | 29 | 141 | 7,24E-19 | 6,57E-17 |
| 10_12318645 | 10 | 12318645 |  | 594 | 326 | 7,61E-19 | 6,89E-17 |
| 1_32266754 | 1 | 32266754 |  | 287 | 541 | 7,84E-19 | 7,09E-17 |
| 6_152708813 | 6 | 152708813 |  | 202 | 422 | 8,00E-19 | 7,22E-17 |
| 7_102788298 | 7 | 102788298 |  | 128 | 312 | 8,24E-19 | 7,42E-17 |
| 7_120971367 | 7 | 120971367 |  | 378 | 172 | 9,03E-19 | 8,12E-17 |
| 12_24903604 | 12 | 24903604 |  | 1041 | 675 | 9,19E-19 | 8,25E-17 |
| 17_36283899 | 17 | 36283899 |  | 640 | 361 | 9,23E-19 | 8,27E-17 |
| 14_149178385 | 14 | 149178385 |  | 355 | 632 | 9,29E-19 | 8,31E-17 |
| 8_12417315 | 8 | 12417315 | ENSSSCG00000028448 | 786 | 473 | 9,70E-19 | 8,67E-17 |
| 5_73857304 | 5 | 73857304 |  | 83 | 239 | 1,17E-18 | 1,04E-16 |
| 2_134987363 | 2 | 134987363 |  | 643 | 364 | 1,18E-18 | 1,05E-16 |
| 4_20283443 | 4 | 20283443 | TAF2 | 232 | 463 | 1,29E-18 | 1,15E-16 |
| 13_149711615 | 13 | 149711615 |  | 707 | 414 | 1,77E-18 | 1,57E-16 |
| 8_3265111 | 8 | 3265111 |  | 586 | 323 | 2,11E-18 | 1,87E-16 |
| 7_34989231 | 7 | 34989231 | HMGA1 | 972 | 623 | 2,17E-18 | 1,91E-16 |
| 8_19874124 | 8 | 19874124 |  | 249 | 90 | 2,17E-18 | 1,92E-16 |
| 2_83138799 | 2 | 83138799 |  | 2523 | 1939 | 2,33E-18 | 2,05E-16 |
| 12_20281665 | 12 | 20281665 |  | 172 | 375 | 2,35E-18 | 2,07E-16 |
| 6_43733956 | 6 | 43733956 |  | 284 | 112 | 2,45E-18 | 2,15E-16 |
| 2_5942383 | 2 | 5942383 |  | 925 | 586 | 2,51E-18 | 2,20E-16 |
| 16_25803163 | 16 | 25803163 |  | 301 | 123 | 2,60E-18 | 2,28E-16 |
| 15_66430664 | 15 | 66430664 | ENSSSCG00000015864 | 1814 | 1325 | 2,60E-18 | 2,28E-16 |
| 5_65642052 | 5 | 65642052 |  | 438 | 216 | 2,63E-18 | 2,29E-16 |
| 14_108788173 | 14 | 108788173 | PAPSS2 | 731 | 434 | 2,80E-18 | 2,44E-16 |
| 10_47234906 | 10 | 47234906 |  | 855 | 531 | 2,89E-18 | 2,52E-16 |
| 17_29455479 | 17 | 29455479 | DSTN; | 1105 | 732 | 3,06E-18 | 2,66E-16 |
| 3_29981139 | 3 | 29981139 |  | 159 | 355 | 3,08E-18 | 2,67E-16 |
| 7_26973855 | 7 | 26973855 | TUBB; | 639 | 364 | 3,14E-18 | 2,71E-16 |
| 14_58841382 | 14 | 58841382 | HEATR1 | 108 | 277 | 3,14E-18 | 2,72E-16 |
| 6_45174994 | 6 | 45174994 |  | 12 | 99 | 3,45E-18 | 2,97E-16 |
| 6_16848513 | 6 | 16848513 | CDH3; | 31 | 142 | 3,54E-18 | 3,05E-16 |
| 14_98580295 | 14 | 98580295 |  | 2750 | 2142 | 3,64E-18 | 3,13E-16 |
| 5_16488749 | 5 | 16488749 |  | 610 | 953 | 3,78E-18 | 3,24E-16 |
| 1_182892047 | 1 | 182892047 | AAGAB | 281 | 111 | 4,10E-18 | 3,52E-16 |
| 6_44968332 | 6 | 44968332 | CYP2B22 | 199 | 412 | 4,50E-18 | 3,85E-16 |
| 13_145020581 | 13 | 145020581 | MUC13A | 342 | 608 | 4,96E-18 | 4,23E-16 |
| 12_24003070 | 12 | 24003070 |  | 1376 | 958 | 4,95E-18 | 4,23E-16 |
| 12_23997646 | 12 | 23997646 | PNPO | 790 | 1173 | 5,19E-18 | 4,42E-16 |
| 7_28045312 | 7 | 28045312 |  | 768 | 465 | 5,43E-18 | 4,62E-16 |
| 6_66639214 | 6 | 66639214 |  | 29 | 137 | 5,48E-18 | 4,65E-16 |
| 17_34561423 | 17 | 34561423 |  | 30 | 139 | 5,68E-18 | 4,81E-16 |
| 15_116212547 | 15 | 116212547 |  | 839 | 521 | 5,87E-18 | 4,97E-16 |
| 1_286605014 | 1 | 286605014 |  | 694 | 408 | 5,94E-18 | 5,02E-16 |
| 14_58844635 | 14 | 58844635 |  | 648 | 373 | 6,29E-18 | 5,30E-16 |
| 7_24641564 | 7 | 24641564 |  | 366 | 168 | 6,39E-18 | 5,37E-16 |
| 6_119074094 | 6 | 119074094 | ENSSSCG00000003753 | 14 | 103 | 6,38E-18 | 5,37E-16 |
| 4_113115322 | 4 | 113115322 |  | 11 | 95 | 7,73E-18 | 6,48E-16 |
| 8_12403657 | 8 | 12403657 |  | 1112 | 742 | 8,10E-18 | 6,79E-16 |
| 6_43234155 | 6 | 43234155 |  | 160 | 41 | 8,36E-18 | 6,99E-16 |
| 10_21632319 | 10 | 21632319 | ENSSSCG00000010884 | 18 | 112 | 8,92E-18 | 7,45E-16 |
| 13_141229923 | 13 | 141229923 |  | 548 | 299 | 9,21E-18 | 7,68E-16 |
| 14_36790651 | 14 | 36790651 |  | 1167 | 788 | 9,85E-18 | 8,20E-16 |
| X_115090286 | X | 115090286 |  | 240 | 467 | 1,00E-17 | 8,32E-16 |
| 2_12842117 | 2 | 12842117 |  | 835 | 520 | 1,05E-17 | 8,69E-16 |
| 8_80738009 | 8 | 80738009 |  | 601 | 339 | 1,05E-17 | 8,69E-16 |
| 14_9500370 | 14 | 9500370 | ADAMDEC1 | 389 | 185 | 1,09E-17 | 8,99E-16 |
| 5_8899156 | 5 | 8899156 | EIF3D | 1034 | 680 | 1,16E-17 | 9,59E-16 |
| 7_35110058 | 7 | 35110058 |  | 242 | 469 | 1,24E-17 | 1,03E-15 |
| 13_15479524 | 13 | 15479524 |  | 462 | 237 | 1,27E-17 | 1,04E-15 |
| 12_43758652 | 12 | 43758652 |  | 1527 | 1090 | 1,31E-17 | 1,08E-15 |
| 14_58843692 | 14 | 58843692 |  | 128 | 304 | 1,34E-17 | 1,10E-15 |
| 7_103573571 | 7 | 103573571 |  | 774 | 473 | 1,38E-17 | 1,13E-15 |
| 6_137327115 | 6 | 137327115 |  | 27 | 131 | 1,38E-17 | 1,13E-15 |
| 6_152708803 | 6 | 152708803 |  | 209 | 422 | 1,58E-17 | 1,29E-15 |
| 12_24902723 | 12 | 24902723 |  | 633 | 365 | 1,85E-17 | 1,51E-15 |
| 3_99313116 | 3 | 99313116 |  | 650 | 378 | 1,87E-17 | 1,52E-15 |
| 16_29451837 | 16 | 29451837 | HMGCS1 | 1425 | 1006 | 1,92E-17 | 1,56E-15 |
| 6_43234154 | 6 | 43234154 |  | 158 | 41 | 2,13E-17 | 1,73E-15 |
| 14_41208716 | 14 | 41208716 | OAS2 | 418 | 207 | 2,23E-17 | 1,81E-15 |
| 17_53183180 | 17 | 53183180 |  | 824 | 1206 | 2,23E-17 | 1,81E-15 |
| 18_50053105 | 18 | 50053105 | HOXA7 | 92 | 246 | 2,38E-17 | 1,92E-15 |
| 12_23441204 | 12 | 23441204 |  | 52 | 178 | 2,51E-17 | 2,03E-15 |
| 8_10837763 | 8 | 10837763 |  | 1137 | 768 | 2,73E-17 | 2,20E-15 |
| 6_70509829 | 6 | 70509829 |  | 138 | 317 | 2,77E-17 | 2,23E-15 |
| 10_38205963 | 10 | 38205963 |  | 1952 | 1459 | 3,21E-17 | 2,58E-15 |
| 6_111620066 | 6 | 111620066 |  | 54 | 181 | 3,26E-17 | 2,61E-15 |
| 6_125312443 | 6 | 125312443 |  | 999 | 656 | 3,26E-17 | 2,61E-15 |
| 2_9703180 | 2 | 9703180 |  | 591 | 335 | 3,35E-17 | 2,68E-15 |
| 1_303017215 | 1 | 303017215 | SPTAN1 | 1752 | 1287 | 3,36E-17 | 2,69E-15 |
| 14_58844426 | 14 | 58844426 |  | 995 | 653 | 3,46E-17 | 2,76E-15 |
| 4_139653900 | 4 | 139653900 | GBP1 | 1389 | 979 | 3,59E-17 | 2,85E-15 |
| 2_136458166 | 2 | 136458166 |  | 1485 | 1060 | 3,64E-17 | 2,89E-15 |
| 6_43736023 | 6 | 43736023 |  | 385 | 185 | 3,68E-17 | 2,92E-15 |
| 16_29452295 | 16 | 29452295 | HMGCS1 | 1085 | 727 | 3,97E-17 | 3,14E-15 |
| 1_91722154 | 1 | 91722154 |  | 915 | 589 | 4,00E-17 | 3,16E-15 |
| 14_52148957 | 14 | 52148957 | SLC5A1 | 808 | 1183 | 4,23E-17 | 3,34E-15 |
| 8_10782780 | 8 | 10782780 |  | 164 | 45 | 4,27E-17 | 3,36E-15 |
| 15_139184280 | 15 | 139184280 |  | 653 | 383 | 4,28E-17 | 3,37E-15 |
| 14_33671046 | 14 | 33671046 |  | 434 | 220 | 4,30E-17 | 3,38E-15 |
| 16_27701408 | 16 | 27701408 |  | 574 | 323 | 4,39E-17 | 3,44E-15 |
| 16_27702060 | 16 | 27702060 |  | 559 | 312 | 4,79E-17 | 3,76E-15 |
| 1_145979762 | 1 | 145979762 |  | 201 | 66 | 4,95E-17 | 3,87E-15 |
| 2_121507621 | 2 | 121507621 | REEP5 | 935 | 606 | 4,98E-17 | 3,89E-15 |
| 14_80626307 | 14 | 80626307 |  | 920 | 594 | 5,08E-17 | 3,97E-15 |
| 7_28043570 | 7 | 28043570 |  | 682 | 406 | 5,21E-17 | 4,06E-15 |
| 12_20283047 | 12 | 20283047 |  | 115 | 280 | 5,45E-17 | 4,24E-15 |
| 2_221641 | 2 | 221641 |  | 272 | 110 | 5,96E-17 | 4,63E-15 |
| 9_146590910 | 9 | 146590910 |  | 1165 | 795 | 6,30E-17 | 4,89E-15 |
| 3_32129908 | 3 | 32129908 | TNFRSF17 | 316 | 139 | 6,36E-17 | 4,93E-15 |
| 12_23970653 | 12 | 23970653 |  | 344 | 158 | 6,61E-17 | 5,11E-15 |
| 7_7768881 | 7 | 7768881 |  | 144 | 35 | 6,67E-17 | 5,15E-15 |
| 18_6549846 | 18 | 6549846 |  | 181 | 55 | 7,04E-17 | 5,43E-15 |
| 7_102789298 | 7 | 102789298 |  | 49 | 170 | 7,79E-17 | 6,00E-15 |
| 5_3358134 | 5 | 3358134 | PACSIN2 | 238 | 89 | 7,85E-17 | 6,04E-15 |
| 14_58843871 | 14 | 58843871 |  | 108 | 268 | 8,17E-17 | 6,27E-15 |
| 2_16506913 | 2 | 16506913 | ENSSSCG00000025402 | 6 | 77 | 8,45E-17 | 6,47E-15 |
| 2_16506912 | 2 | 16506912 | ENSSSCG00000025402 | 6 | 77 | 8,45E-17 | 6,47E-15 |
| 2_13408586 | 2 | 13408586 | TNKS1BP1 | 546 | 304 | 8,62E-17 | 6,59E-15 |
| 4_77293029 | 4 | 77293029 | GGH | 1089 | 734 | 9,02E-17 | 6,88E-15 |
| 6_13128565 | 6 | 13128565 |  | 1119 | 759 | 9,63E-17 | 7,34E-15 |
| 6_70495018 | 6 | 70495018 |  | 220 | 431 | 1,01E-16 | 7,68E-15 |
| 14_88971270 | 14 | 88971270 |  | 112 | 20 | 1,04E-16 | 7,87E-15 |
| 13_133979507 | 13 | 133979507 | EIF4A2 | 1584 | 1150 | 1,06E-16 | 8,01E-15 |
| 4_38941810 | 4 | 38941810 |  | 1043 | 697 | 1,06E-16 | 8,03E-15 |
| 2_157504017 | 2 | 157504017 |  | 248 | 96 | 1,23E-16 | 9,31E-15 |
| 5_82654323 | 5 | 82654323 |  | 100 | 254 | 1,39E-16 | 1,05E-14 |
| 9_3723081 | 9 | 3723081 | ENSSSCG00000014618 | 11 | 90 | 1,42E-16 | 1,07E-14 |
| 2_50268101 | 2 | 50268101 |  | 60 | 188 | 1,49E-16 | 1,12E-14 |
| 17_34917938 | 17 | 34917938 |  | 25 | 122 | 1,58E-16 | 1,19E-14 |
| 13_149716767 | 13 | 149716767 |  | 819 | 518 | 1,73E-16 | 1,30E-14 |
| 14_138042 | 14 | 138042 |  | 394 | 195 | 1,76E-16 | 1,32E-14 |
| 7_24653157 | 7 | 24653157 |  | 72 | 208 | 1,78E-16 | 1,33E-14 |
| 1_303414745 | 1 | 303414745 |  | 443 | 724 | 1,78E-16 | 1,33E-14 |
| 14_31685333 | 14 | 31685333 |  | 278 | 116 | 1,88E-16 | 1,40E-14 |
| 6_57852921 | 6 | 57852921 |  | 560 | 871 | 1,92E-16 | 1,43E-14 |
| 2_11810898 | 2 | 11810898 | MPEG1 | 593 | 342 | 1,95E-16 | 1,45E-14 |
| 18_55402129 | 18 | 55402129 |  | 516 | 284 | 1,96E-16 | 1,45E-14 |
| 10_65809960 | 10 | 65809960 | ECHDC3 | 284 | 120 | 1,95E-16 | 1,45E-14 |
| 5_18080597 | 5 | 18080597 | ENSSSCG00000000239 | 296 | 128 | 2,04E-16 | 1,51E-14 |
| 2_134981251 | 2 | 134981251 | ENSSSCG00000014247 | 490 | 265 | 2,16E-16 | 1,60E-14 |
| 15_34678513 | 15 | 34678513 | MKI67 | 677 | 1014 | 2,45E-16 | 1,81E-14 |
| 12_25383320 | 12 | 25383320 | ABI3 | 626 | 368 | 2,46E-16 | 1,81E-14 |
| 7_24653147 | 7 | 24653147 |  | 69 | 202 | 2,62E-16 | 1,93E-14 |
| 15_28583124 | 15 | 28583124 | DBI | 978 | 1375 | 2,77E-16 | 2,04E-14 |
| 7_35110064 | 7 | 35110064 |  | 243 | 459 | 2,85E-16 | 2,10E-14 |
| 2_134987303 | 2 | 134987303 |  | 695 | 422 | 2,86E-16 | 2,10E-14 |
| 8_12417379 | 8 | 12417379 | ENSSSCG00000028448 | 1008 | 673 | 3,01E-16 | 2,20E-14 |
| 13_28954253 | 13 | 28954253 |  | 178 | 368 | 3,04E-16 | 2,23E-14 |
| 3_115787249 | 3 | 115787249 |  | 612 | 933 | 3,08E-16 | 2,25E-14 |
| 9_67492433 | 9 | 67492433 | ENSSSCG00000025707 | 247 | 97 | 3,18E-16 | 2,32E-14 |
| 7_102787801 | 7 | 102787801 |  | 123 | 287 | 3,33E-16 | 2,43E-14 |
| 2_9703243 | 2 | 9703243 |  | 716 | 439 | 3,35E-16 | 2,44E-14 |
| 2_141885943 | 2 | 141885943 |  | 1688 | 1246 | 3,44E-16 | 2,50E-14 |
| 4_121730569 | 4 | 121730569 |  | 59 | 184 | 3,86E-16 | 2,80E-14 |
| 17_53822373 | 17 | 53822373 | MMP9 | 306 | 136 | 3,94E-16 | 2,86E-14 |
| 16_25801596 | 16 | 25801596 |  | 297 | 130 | 3,99E-16 | 2,89E-14 |
| 14_12473364 | 14 | 12473364 | EPHX2 | 50 | 168 | 4,14E-16 | 3,00E-14 |
| 1_302314311 | 1 | 302314311 |  | 463 | 247 | 4,24E-16 | 3,06E-14 |
| 12_1165434 | 12 | 1165434 | BETA-ACTIN | 787 | 496 | 4,26E-16 | 3,07E-14 |
| 8_144530481 | 8 | 144530481 | ENSSSCG00000009239 | 147 | 322 | 4,29E-16 | 3,09E-14 |
| 6_131561676 | 6 | 131561676 | ZRANB2; | 905 | 591 | 4,59E-16 | 3,30E-14 |
| 6_88651459 | 6 | 88651459 | ENSSSCG00000003664 | 622 | 367 | 4,60E-16 | 3,31E-14 |
| 6_119273223 | 6 | 119273223 | MCOLN2 | 155 | 43 | 4,69E-16 | 3,36E-14 |
| 6_13128502 | 6 | 13128502 |  | 1163 | 803 | 4,70E-16 | 3,37E-14 |
| 14_41208718 | 14 | 41208718 | OAS2 | 413 | 211 | 4,75E-16 | 3,40E-14 |
| 13_32799185 | 13 | 32799185 |  | 16 | 100 | 4,96E-16 | 3,54E-14 |
| 2_65158743 | 2 | 65158743 |  | 668 | 403 | 5,14E-16 | 3,66E-14 |
| 7_83479750 | 7 | 83479750 | TMEM55B | 249 | 465 | 5,14E-16 | 3,67E-14 |
| 9_67490137 | 9 | 67490137 | ENSSSCG00000025707 | 185 | 60 | 5,22E-16 | 3,71E-14 |
| 4_139594281 | 4 | 139594281 | GBP4 | 898 | 586 | 5,37E-16 | 3,82E-14 |
| 14_149178338 | 14 | 149178338 |  | 497 | 787 | 5,53E-16 | 3,92E-14 |
| 6_75388707 | 6 | 75388707 | FUCA1 | 958 | 635 | 5,72E-16 | 4,05E-14 |
| 7_29188637 | 7 | 29188637 |  | 608 | 357 | 5,81E-16 | 4,11E-14 |
| 7_2014497 | 7 | 2014497 |  | 143 | 37 | 6,63E-16 | 4,68E-14 |
| 10_37497982 | 10 | 37497982 | BAG1 | 18 | 104 | 6,78E-16 | 4,78E-14 |
| 6_43733781 | 6 | 43733781 |  | 371 | 182 | 6,82E-16 | 4,80E-14 |
| 4_122128232 | 4 | 122128232 |  | 631 | 952 | 7,02E-16 | 4,94E-14 |
| 3_119782506 | 3 | 119782506 | HADHA; | 895 | 585 | 7,55E-16 | 5,31E-14 |
| 1_24149773 | 1 | 24149773 | FUCA2 | 19 | 106 | 7,70E-16 | 5,40E-14 |
| 14_98578478 | 14 | 98578478 |  | 1239 | 869 | 7,79E-16 | 5,46E-14 |
| 10_21621732 | 10 | 21621732 | ENSSSCG00000010884 | 60 | 184 | 7,90E-16 | 5,53E-14 |
| 8_92866442 | 8 | 92866442 | MAML3 | 30 | 129 | 7,92E-16 | 5,53E-14 |
| 10_26331253 | 10 | 26331253 | PTPRC | 662 | 400 | 8,31E-16 | 5,80E-14 |
| 6_58008138 | 6 | 58008138 | SDF4 | 1176 | 817 | 8,94E-16 | 6,23E-14 |
| 4_19716428 | 4 | 19716428 |  | 477 | 260 | 1,11E-15 | 7,71E-14 |
| 9_31847574 | 9 | 31847574 |  | 545 | 311 | 1,11E-15 | 7,75E-14 |
| 9_67490140 | 9 | 67490140 | ENSSSCG00000025707 | 176 | 56 | 1,25E-15 | 8,66E-14 |
| 14_58843802 | 14 | 58843802 |  | 104 | 254 | 1,30E-15 | 9,03E-14 |
| 7_34508581 | 7 | 34508581 | ITPR3 | 1440 | 1042 | 1,40E-15 | 9,68E-14 |
| 13_156316390 | 13 | 156316390 |  | 544 | 841 | 1,42E-15 | 9,80E-14 |
| 17_46910683 | 17 | 46910683 |  | 16 | 98 | 1,48E-15 | 1,02E-13 |
| 6_119273209 | 6 | 119273209 | MCOLN2 | 172 | 54 | 1,55E-15 | 1,07E-13 |
| 11_20025491 | 11 | 20025491 |  | 261 | 109 | 1,66E-15 | 1,14E-13 |
| 9_150092247 | 9 | 150092247 |  | 182 | 60 | 1,80E-15 | 1,24E-13 |
| 6_40112171 | 6 | 40112171 |  | 652 | 395 | 1,85E-15 | 1,27E-13 |
| 6_14032604 | 6 | 14032604 |  | 317 | 147 | 2,10E-15 | 1,44E-13 |
| 14_92224223 | 14 | 92224223 | C10orf99 | 101 | 248 | 2,10E-15 | 1,44E-13 |
| 7_5315383 | 7 | 5315383 | BMP6 | 1154 | 803 | 2,14E-15 | 1,47E-13 |
| 10_64846511 | 10 | 64846511 |  | 170 | 350 | 2,20E-15 | 1,51E-13 |
| 14_78507839 | 14 | 78507839 |  | 493 | 274 | 2,28E-15 | 1,55E-13 |
| 6_70509805 | 6 | 70509805 |  | 152 | 324 | 2,28E-15 | 1,56E-13 |
| 4_110276642 | 4 | 110276642 |  | 38 | 142 | 2,52E-15 | 1,72E-13 |
| 6_152707629 | 6 | 152707629 |  | 777 | 495 | 2,55E-15 | 1,74E-13 |
| 2_13002750 | 2 | 13002750 | UBE2L6 | 255 | 106 | 2,70E-15 | 1,84E-13 |
| 7_48120616 | 7 | 48120616 | ENSSSCG00000001720 | 181 | 60 | 2,72E-15 | 1,85E-13 |
| 2_44644972 | 2 | 44644972 | USH1C | 1287 | 916 | 2,76E-15 | 1,87E-13 |
| 12_24901472 | 12 | 24901472 |  | 706 | 439 | 2,87E-15 | 1,94E-13 |
| 13_38070196 | 13 | 38070196 |  | 141 | 307 | 3,10E-15 | 2,10E-13 |
| 10_37497861 | 10 | 37497861 | BAG1 | 46 | 156 | 3,41E-15 | 2,30E-13 |
| 8_139941103 | 8 | 139941103 | PIGY | 421 | 222 | 3,51E-15 | 2,37E-13 |
| 6_119272657 | 6 | 119272657 | MCOLN2 | 337 | 162 | 3,53E-15 | 2,38E-13 |
| 6_152322127 | 6 | 152322127 |  | 624 | 376 | 4,13E-15 | 2,78E-13 |
| 12_21902594 | 12 | 21902594 |  | 501 | 782 | 4,22E-15 | 2,83E-13 |
| 16_78290111 | 16 | 78290111 | GPX3 | 43 | 150 | 4,40E-15 | 2,95E-13 |
| X_41168428 | X | 41168428 | DDX3X | 11 | 84 | 4,52E-15 | 3,03E-13 |
| 7_36141794 | 7 | 36141794 |  | 186 | 370 | 4,78E-15 | 3,19E-13 |
| 5_38418090 | 5 | 38418090 | TMEM19 | 211 | 79 | 4,81E-15 | 3,21E-13 |
| 9_136806168 | 9 | 136806168 | ENSSSCG00000015556 | 1183 | 1596 | 4,89E-15 | 3,26E-13 |
| 2_134987709 | 2 | 134987709 |  | 721 | 453 | 5,03E-15 | 3,35E-13 |
| 2_6453168 | 2 | 6453168 |  | 155 | 46 | 5,28E-15 | 3,51E-13 |
| 6_43733821 | 6 | 43733821 |  | 298 | 136 | 5,28E-15 | 3,51E-13 |
| 6_119073833 | 6 | 119073833 |  | 10 | 81 | 5,90E-15 | 3,92E-13 |
| 7_120963632 | 7 | 120963632 | CHGA | 5 | 67 | 6,39E-15 | 4,23E-13 |
| 8_144530862 | 8 | 144530862 | ENSSSCG00000009239 | 842 | 1193 | 7,39E-15 | 4,89E-13 |
| 2_77822774 | 2 | 77822774 |  | 884 | 586 | 7,69E-15 | 5,08E-13 |
| 18_53427196 | 18 | 53427196 |  | 1167 | 1574 | 7,92E-15 | 5,23E-13 |
| 14_58851001 | 14 | 58851001 |  | 11 | 83 | 8,00E-15 | 5,28E-13 |
| 1_3691558 | 1 | 3691558 |  | 946 | 637 | 8,13E-15 | 5,35E-13 |
| 10_22597797 | 10 | 22597797 |  | 61 | 180 | 8,16E-15 | 5,37E-13 |
| 2_125335655 | 2 | 125335655 | COMMD10 | 90 | 227 | 8,35E-15 | 5,49E-13 |
| 4_121322689 | 4 | 121322689 |  | 1542 | 1140 | 8,67E-15 | 5,69E-13 |
| 7_14563353 | 7 | 14563353 |  | 80 | 211 | 8,81E-15 | 5,78E-13 |
| 17_34562023 | 17 | 34562023 |  | 29 | 122 | 9,02E-15 | 5,91E-13 |
| 9_3619027 | 9 | 3619027 |  | 165 | 338 | 9,49E-15 | 6,21E-13 |
| 6_43733820 | 6 | 43733820 |  | 296 | 136 | 1,00E-14 | 6,53E-13 |
| 8_145146402 | 8 | 145146402 |  | 309 | 145 | 1,04E-14 | 6,75E-13 |
| 4_139599569 | 4 | 139599569 | GBP4 | 1016 | 696 | 1,06E-14 | 6,90E-13 |
| 2_157502955 | 2 | 157502955 |  | 191 | 68 | 1,07E-14 | 6,95E-13 |
| 14_16203377 | 14 | 16203377 |  | 604 | 904 | 1,12E-14 | 7,28E-13 |
| 14_14140269 | 14 | 14140269 |  | 650 | 400 | 1,16E-14 | 7,52E-13 |
| 6_119078593 | 6 | 119078593 | ENSSSCG00000003753 | 21 | 105 | 1,25E-14 | 8,13E-13 |
| 1_91723028 | 1 | 91723028 |  | 125 | 31 | 1,40E-14 | 9,03E-13 |
| 1_280430303 | 1 | 280430303 |  | 200 | 74 | 1,46E-14 | 9,43E-13 |
| 15_122431144 | 15 | 122431144 |  | 234 | 432 | 1,47E-14 | 9,48E-13 |
| 10_12317786 | 10 | 12317786 |  | 227 | 91 | 1,47E-14 | 9,49E-13 |
| 10_38214395 | 10 | 38214395 |  | 1511 | 1117 | 1,59E-14 | 1,02E-12 |
| X_112842723 | X | 112842723 |  | 48 | 156 | 1,59E-14 | 1,03E-12 |
| 2_10740907 | 2 | 10740907 |  | 8 | 74 | 1,65E-14 | 1,06E-12 |
| 2_134961237 | 2 | 134961237 | ENSSSCG00000014247 | 374 | 192 | 1,66E-14 | 1,07E-12 |
| 6_52803887 | 6 | 52803887 | ENSSSCG00000003252 | 303 | 142 | 1,73E-14 | 1,11E-12 |
| 5_82654001 | 5 | 82654001 |  | 64 | 183 | 1,84E-14 | 1,18E-12 |
| 4_121730577 | 4 | 121730577 |  | 75 | 201 | 1,85E-14 | 1,18E-12 |
| 14_58850993 | 14 | 58850993 |  | 10 | 79 | 1,87E-14 | 1,20E-12 |
| 4_104764325 | 4 | 104764325 |  | 41 | 143 | 1,96E-14 | 1,25E-12 |
| 16_4339638 | 16 | 4339638 |  | 171 | 57 | 2,02E-14 | 1,29E-12 |
| 7_41640914 | 7 | 41640914 |  | 69 | 191 | 2,03E-14 | 1,29E-12 |
| 13_24858592 | 13 | 24858592 |  | 724 | 461 | 2,14E-14 | 1,36E-12 |
| 2_8653818 | 2 | 8653818 |  | 122 | 273 | 2,15E-14 | 1,36E-12 |
| 2_18637753 | 2 | 18637753 | SYT13 | 270 | 120 | 2,17E-14 | 1,37E-12 |
| 13_157419709 | 13 | 157419709 |  | 309 | 530 | 2,19E-14 | 1,38E-12 |
| 2_134987660 | 2 | 134987660 |  | 690 | 434 | 2,19E-14 | 1,39E-12 |
| 1_3681211 | 1 | 3681211 |  | 261 | 114 | 2,20E-14 | 1,39E-12 |
| 13_149712246 | 13 | 149712246 |  | 666 | 415 | 2,21E-14 | 1,39E-12 |
| 10_12317508 | 10 | 12317508 |  | 552 | 326 | 2,25E-14 | 1,42E-12 |
| 7_5019616 | 7 | 5019616 | DSP | 905 | 608 | 2,28E-14 | 1,43E-12 |
| 4_121594462 | 4 | 121594462 |  | 13 | 86 | 2,29E-14 | 1,44E-12 |
| 1_61543678 | 1 | 61543678 |  | 30 | 122 | 2,31E-14 | 1,45E-12 |
| 11_20025529 | 11 | 20025529 |  | 81 | 210 | 2,32E-14 | 1,45E-12 |
| 6_124828377 | 6 | 124828377 |  | 549 | 324 | 2,48E-14 | 1,55E-12 |
| 2_156119672 | 2 | 156119672 |  | 282 | 494 | 2,51E-14 | 1,57E-12 |
| 6_152214701 | 6 | 152214701 |  | 321 | 545 | 2,54E-14 | 1,58E-12 |
| 9_3619032 | 9 | 3619032 |  | 191 | 371 | 2,62E-14 | 1,64E-12 |
| 14_14139488 | 14 | 14139488 |  | 375 | 194 | 2,74E-14 | 1,71E-12 |
| 2_134987440 | 2 | 134987440 |  | 750 | 483 | 2,86E-14 | 1,78E-12 |
| 2_18637751 | 2 | 18637751 | SYT13 | 269 | 120 | 3,01E-14 | 1,87E-12 |
| 4_97327233 | 4 | 97327233 |  | 1468 | 1084 | 3,06E-14 | 1,90E-12 |
| 13_156322269 | 13 | 156322269 |  | 669 | 419 | 3,40E-14 | 2,11E-12 |
| 6_34524545 | 6 | 34524545 |  | 537 | 316 | 3,60E-14 | 2,23E-12 |
| 2_9352576 | 2 | 9352576 |  | 31 | 123 | 3,60E-14 | 2,23E-12 |
| 16_29023398 | 16 | 29023398 |  | 1731 | 1313 | 3,74E-14 | 2,31E-12 |
| 3_59720486 | 3 | 59720486 |  | 24 | 109 | 3,89E-14 | 2,40E-12 |
| 6_52524909 | 6 | 52524909 |  | 27 | 115 | 3,94E-14 | 2,43E-12 |
| 6_81047762 | 6 | 81047762 |  | 111 | 25 | 3,94E-14 | 2,43E-12 |
| 6_50308783 | 6 | 50308783 | RPL13A | 26 | 113 | 3,96E-14 | 2,43E-12 |
| 4_102641736 | 4 | 102641736 |  | 541 | 820 | 3,98E-14 | 2,44E-12 |
| 2_5813818 | 2 | 5813818 |  | 152 | 47 | 4,16E-14 | 2,55E-12 |
| 8_140714914 | 8 | 140714914 | HSD17B11 | 349 | 579 | 4,17E-14 | 2,56E-12 |
| 8_117260884 | 8 | 117260884 |  | 300 | 142 | 4,40E-14 | 2,69E-12 |
| 13_131634344 | 13 | 131634344 | PSMD2 | 18 | 96 | 4,72E-14 | 2,89E-12 |
| 2_8401828 | 2 | 8401828 | BSCL2 | 18 | 96 | 4,72E-14 | 2,89E-12 |
| 9_146592044 | 9 | 146592044 |  | 344 | 572 | 4,74E-14 | 2,89E-12 |
| 6_78608605 | 6 | 78608605 | SMPDL3B | 494 | 284 | 4,76E-14 | 2,90E-12 |
| 6_52524797 | 6 | 52524797 |  | 19 | 98 | 5,14E-14 | 3,13E-12 |
| 13_20262884 | 13 | 20262884 |  | 322 | 543 | 5,45E-14 | 3,31E-12 |
| 18_49331605 | 18 | 49331605 |  | 100 | 20 | 5,51E-14 | 3,35E-12 |
| 12_27116727 | 12 | 27116727 |  | 70 | 190 | 5,59E-14 | 3,39E-12 |
| 1_303403728 | 1 | 303403728 |  | 180 | 353 | 5,64E-14 | 3,42E-12 |
| 9_74195834 | 9 | 74195834 | ENSSSCG00000015663 | 237 | 100 | 5,76E-14 | 3,49E-12 |
| 3_121940357 | 3 | 121940357 |  | 180 | 64 | 6,00E-14 | 3,63E-12 |
| 12_24896135 | 12 | 24896135 |  | 1521 | 1134 | 6,17E-14 | 3,73E-12 |
| 3_99311938 | 3 | 99311938 |  | 51 | 158 | 6,24E-14 | 3,76E-12 |
| 15_134325918 | 15 | 134325918 |  | 131 | 36 | 6,98E-14 | 4,21E-12 |
| 6_74616549 | 6 | 74616549 | C1QC | 1135 | 805 | 7,03E-14 | 4,23E-12 |
| 1_286624115 | 1 | 286624115 |  | 556 | 333 | 7,18E-14 | 4,32E-12 |
| 7_21021362 | 7 | 21021362 |  | 93 | 17 | 7,24E-14 | 4,34E-12 |
| 2_5944376 | 2 | 5944376 |  | 807 | 533 | 7,24E-14 | 4,34E-12 |
| 4_129752437 | 4 | 129752437 |  | 94 | 227 | 7,65E-14 | 4,58E-12 |
| 4_121376062 | 4 | 121376062 |  | 45 | 147 | 7,82E-14 | 4,68E-12 |
| 15_133507355 | 15 | 133507355 | VIL1 | 1474 | 1095 | 7,95E-14 | 4,75E-12 |
| 7_48120509 | 7 | 48120509 | ENSSSCG00000001720 | 236 | 100 | 8,12E-14 | 4,85E-12 |
| 6_147313318 | 6 | 147313318 |  | 353 | 581 | 8,37E-14 | 4,99E-12 |
| 10_64846499 | 10 | 64846499 |  | 166 | 332 | 8,46E-14 | 5,04E-12 |
| 14_9511939 | 14 | 9511939 | ADAMDEC1 | 19 | 97 | 8,63E-14 | 5,13E-12 |
| 4_91145251 | 4 | 91145251 | CREG1 | 692 | 441 | 8,83E-14 | 5,25E-12 |
| 7_5315236 | 7 | 5315236 | BMP6 | 1215 | 874 | 8,97E-14 | 5,33E-12 |
| 6_88402085 | 6 | 88402085 | MACF1 | 728 | 470 | 9,08E-14 | 5,39E-12 |
| 8_109609183 | 8 | 109609183 | ANXA5 | 503 | 293 | 9,29E-14 | 5,50E-12 |
| 1_39577522 | 1 | 39577522 | ENSSSCG00000004216 | 21 | 101 | 9,68E-14 | 5,73E-12 |
| 18_50031954 | 18 | 50031954 | HOXA10 | 448 | 700 | 1,03E-13 | 6,06E-12 |
| 2_5942429 | 2 | 5942429 |  | 1017 | 708 | 1,04E-13 | 6,16E-12 |
| 2_28657161 | 2 | 28657161 |  | 298 | 509 | 1,05E-13 | 6,20E-12 |
| 14_116530108 | 14 | 116530108 |  | 0 | 44 | 1,14E-13 | 6,70E-12 |
| 13_215061182 | 13 | 215061182 |  | 235 | 100 | 1,14E-13 | 6,73E-12 |
| 16_27055922 | 16 | 27055922 |  | 139 | 41 | 1,15E-13 | 6,74E-12 |
| 6_58279571 | 6 | 58279571 |  | 456 | 258 | 1,17E-13 | 6,89E-12 |
| 6_57887616 | 6 | 57887616 |  | 128 | 35 | 1,18E-13 | 6,89E-12 |
| 3_58239934 | 3 | 58239934 |  | 663 | 419 | 1,18E-13 | 6,94E-12 |
| 6_52543668 | 6 | 52543668 |  | 13 | 83 | 1,20E-13 | 7,03E-12 |
| 1_303403729 | 1 | 303403729 |  | 182 | 353 | 1,24E-13 | 7,23E-12 |
| 3_41660237 | 3 | 41660237 | NME3 | 486 | 281 | 1,27E-13 | 7,39E-12 |
| 2_20061784 | 2 | 20061784 |  | 1043 | 731 | 1,34E-13 | 7,78E-12 |
| 14_41208611 | 14 | 41208611 | OAS2 | 414 | 227 | 1,37E-13 | 8,01E-12 |
| 6_141454842 | 6 | 141454842 |  | 202 | 79 | 1,38E-13 | 8,01E-12 |
| 5_9103312 | 5 | 9103312 |  | 434 | 242 | 1,41E-13 | 8,18E-12 |
| 3_99315043 | 3 | 99315043 |  | 216 | 88 | 1,41E-13 | 8,20E-12 |
| 2_77621080 | 2 | 77621080 | CNN2 | 919 | 628 | 1,42E-13 | 8,22E-12 |
| 2_8701234 | 2 | 8701234 |  | 168 | 333 | 1,43E-13 | 8,26E-12 |
| 2_83139633 | 2 | 83139633 |  | 2403 | 2944 | 1,46E-13 | 8,43E-12 |
| 2_221569 | 2 | 221569 |  | 320 | 536 | 1,50E-13 | 8,66E-12 |
| 11_15771851 | 11 | 15771851 |  | 668 | 424 | 1,54E-13 | 8,88E-12 |
| 6_74879911 | 6 | 74879911 | EPHB2 | 255 | 114 | 1,60E-13 | 9,20E-12 |
| 15_134325921 | 15 | 134325921 |  | 131 | 37 | 1,60E-13 | 9,20E-12 |
| 4_101219762 | 4 | 101219762 |  | 85 | 211 | 1,60E-13 | 9,20E-12 |
| 6_16469159 | 6 | 16469159 |  | 327 | 164 | 1,60E-13 | 9,20E-12 |
| 12_5258691 | 12 | 5258691 | EXOC7 | 363 | 190 | 1,65E-13 | 9,49E-12 |
| 17_41820796 | 17 | 41820796 |  | 314 | 155 | 1,75E-13 | 1,01E-11 |
| 9_127842869 | 9 | 127842869 |  | 91 | 220 | 1,77E-13 | 1,01E-11 |
| 13_144183152 | 13 | 144183152 |  | 543 | 326 | 1,77E-13 | 1,01E-11 |
| 10_37505790 | 10 | 37505790 |  | 41 | 138 | 1,78E-13 | 1,01E-11 |
| 10_47234419 | 10 | 47234419 |  | 651 | 411 | 1,77E-13 | 1,01E-11 |
| 1_302294032 | 1 | 302294032 |  | 651 | 411 | 1,77E-13 | 1,01E-11 |
| 1_302805663 | 1 | 302805663 |  | 321 | 160 | 1,78E-13 | 1,02E-11 |
| 7_7768903 | 7 | 7768903 |  | 127 | 35 | 1,85E-13 | 1,05E-11 |
| 4_15246299 | 4 | 15246299 | CU407238.7 | 483 | 280 | 1,90E-13 | 1,08E-11 |
| 7_130866946 | 7 | 130866946 |  | 325 | 163 | 1,90E-13 | 1,08E-11 |
| 3_119782551 | 3 | 119782551 | HADHA; | 870 | 589 | 1,94E-13 | 1,10E-11 |
| 6_44978723 | 6 | 44978723 | CYP2B22 | 156 | 315 | 1,98E-13 | 1,12E-11 |
| 4_121890558 | 4 | 121890558 |  | 305 | 149 | 2,03E-13 | 1,15E-11 |
| 1_61743471 | 1 | 61743471 |  | 17 | 91 | 2,08E-13 | 1,17E-11 |
| 6_43734025 | 6 | 43734025 |  | 340 | 174 | 2,11E-13 | 1,19E-11 |
| 7_29188605 | 7 | 29188605 |  | 415 | 229 | 2,12E-13 | 1,20E-11 |
| 5_9103381 | 5 | 9103381 |  | 423 | 235 | 2,14E-13 | 1,21E-11 |
| 4_122128108 | 4 | 122128108 |  | 720 | 1027 | 2,14E-13 | 1,21E-11 |
| 8_92875346 | 8 | 92875346 |  | 1248 | 1643 | 2,15E-13 | 1,21E-11 |
| 4_121322685 | 4 | 121322685 |  | 1413 | 1049 | 2,32E-13 | 1,30E-11 |
| 3_99313727 | 3 | 99313727 |  | 153 | 50 | 2,44E-13 | 1,37E-11 |
| 18_6656204 | 18 | 6656204 | TMEM176B | 496 | 755 | 2,48E-13 | 1,39E-11 |
| 4_139601969 | 4 | 139601969 |  | 91 | 219 | 2,51E-13 | 1,40E-11 |
| 1_61543737 | 1 | 61543737 |  | 29 | 115 | 2,55E-13 | 1,43E-11 |
| 8_12332510 | 8 | 12332510 |  | 663 | 422 | 2,56E-13 | 1,43E-11 |
| 10_21621763 | 10 | 21621763 | ENSSSCG00000010884 | 59 | 168 | 2,61E-13 | 1,46E-11 |
| 4_95056680 | 4 | 95056680 |  | 101 | 22 | 2,74E-13 | 1,53E-11 |
| 1_302314371 | 1 | 302314371 |  | 641 | 405 | 2,95E-13 | 1,64E-11 |
| 2_140610440 | 2 | 140610440 |  | 12 | 79 | 2,99E-13 | 1,66E-11 |
| 6_52655158 | 6 | 52655158 |  | 58 | 166 | 2,99E-13 | 1,66E-11 |
| 9_127842870 | 9 | 127842870 |  | 92 | 220 | 3,02E-13 | 1,67E-11 |
| 3_135246450 | 3 | 135246450 | ITGB1BP1 | 1071 | 759 | 3,16E-13 | 1,75E-11 |
| 3_119782443 | 3 | 119782443 | HADHA; | 788 | 524 | 3,21E-13 | 1,78E-11 |
| 3_125990504 | 3 | 125990504 |  | 1564 | 1182 | 3,28E-13 | 1,82E-11 |
| 13_149711919 | 13 | 149711919 |  | 673 | 431 | 3,29E-13 | 1,82E-11 |
| 16_79301669 | 16 | 79301669 |  | 337 | 173 | 3,33E-13 | 1,84E-11 |
| 6_83224288 | 6 | 83224288 |  | 1807 | 1395 | 3,51E-13 | 1,94E-11 |
| 2_146615080 | 2 | 146615080 | CTNNA1 | 886 | 605 | 3,53E-13 | 1,94E-11 |
| 14_33671048 | 14 | 33671048 |  | 458 | 263 | 3,63E-13 | 2,00E-11 |
| 5_3358146 | 5 | 3358146 | PACSIN2 | 492 | 289 | 3,63E-13 | 2,00E-11 |
| 12_5244073 | 12 | 5244073 | EXOC7 | 313 | 523 | 3,70E-13 | 2,03E-11 |
| 12_24901455 | 12 | 24901455 |  | 621 | 390 | 3,74E-13 | 2,05E-11 |
| 17_36999951 | 17 | 36999951 |  | 612 | 894 | 3,82E-13 | 2,09E-11 |
| X_11347881 | X | 11347881 | OFD1 | 353 | 185 | 3,91E-13 | 2,14E-11 |
| 6_119078602 | 6 | 119078602 | ENSSSCG00000003753 | 20 | 96 | 4,25E-13 | 2,32E-11 |
| 16_25813572 | 16 | 25813572 |  | 336 | 173 | 4,41E-13 | 2,41E-11 |
| 3_59514080 | 3 | 59514080 |  | 58 | 165 | 4,44E-13 | 2,42E-11 |
| 2_8665198 | 2 | 8665198 |  | 48 | 148 | 4,68E-13 | 2,55E-11 |
| 13_15479507 | 13 | 15479507 |  | 441 | 251 | 4,84E-13 | 2,63E-11 |
| 10_13582906 | 10 | 13582906 | MIA3 | 686 | 443 | 4,84E-13 | 2,64E-11 |
| 14_124925033 | 14 | 124925033 |  | 60 | 168 | 5,01E-13 | 2,72E-11 |
| 8_1292521 | 8 | 1292521 |  | 286 | 138 | 5,50E-13 | 2,99E-11 |
| 14_41197377 | 14 | 41197377 | OAS2 | 303 | 150 | 5,59E-13 | 3,03E-11 |
| 14_79264783 | 14 | 79264783 |  | 364 | 194 | 5,60E-13 | 3,04E-11 |
| 14_118624361 | 14 | 118624361 |  | 504 | 300 | 6,14E-13 | 3,32E-11 |
| 6_52473361 | 6 | 52473361 |  | 201 | 81 | 6,17E-13 | 3,34E-11 |
| 12_15091657 | 12 | 15091657 | SMARCD2 | 621 | 392 | 6,30E-13 | 3,40E-11 |
| 6_31866136 | 6 | 31866136 |  | 811 | 546 | 6,52E-13 | 3,52E-11 |
| 9_7450742 | 9 | 7450742 |  | 76 | 193 | 6,57E-13 | 3,54E-11 |
| 7_1954427 | 7 | 1954427 | BPHL | 19 | 93 | 6,75E-13 | 3,63E-11 |
| 7_2014536 | 7 | 2014536 |  | 93 | 19 | 6,75E-13 | 3,63E-11 |
| 2_124815698 | 2 | 124815698 |  | 63 | 172 | 7,12E-13 | 3,83E-11 |
| 2_221636 | 2 | 221636 |  | 57 | 162 | 7,55E-13 | 4,06E-11 |
| 6_58277671 | 6 | 58277671 |  | 1873 | 1459 | 7,85E-13 | 4,21E-11 |
| 6_52589450 | 6 | 52589450 |  | 43 | 138 | 8,17E-13 | 4,38E-11 |
| 15_16062133 | 15 | 16062133 |  | 342 | 179 | 8,34E-13 | 4,46E-11 |
| 4_43122939 | 4 | 43122939 |  | 276 | 132 | 8,43E-13 | 4,51E-11 |
| 12_44877365 | 12 | 44877365 | UTP6 | 155 | 53 | 8,62E-13 | 4,60E-11 |
| 7_48119642 | 7 | 48119642 | ENSSSCG00000001720 | 173 | 64 | 9,05E-13 | 4,83E-11 |
| 7_4676622 | 7 | 4676622 |  | 4 | 56 | 9,08E-13 | 4,84E-11 |
| 7_35769178 | 7 | 35769178 |  | 332 | 172 | 9,19E-13 | 4,89E-11 |
| 16_77975800 | 16 | 77975800 |  | 783 | 525 | 1,01E-12 | 5,39E-11 |
| 4_110200993 | 4 | 110200993 | FMO5 | 734 | 485 | 1,02E-12 | 5,43E-11 |
| 8_31753628 | 8 | 31753628 | FAM114A1 | 258 | 120 | 1,03E-12 | 5,48E-11 |
| 14_122674699 | 14 | 122674699 |  | 875 | 601 | 1,03E-12 | 5,49E-11 |
| 10_33263941 | 10 | 33263941 |  | 426 | 242 | 1,04E-12 | 5,51E-11 |
| 1_269076739 | 1 | 269076739 |  | 187 | 73 | 1,05E-12 | 5,54E-11 |
| 4_91145169 | 4 | 91145169 | CREG1 | 730 | 482 | 1,08E-12 | 5,74E-11 |
| 2_11810529 | 2 | 11810529 | MPEG1 | 763 | 509 | 1,10E-12 | 5,83E-11 |
| 7_48120662 | 7 | 48120662 | ENSSSCG00000001720 | 156 | 54 | 1,13E-12 | 5,96E-11 |
| 4_92673646 | 4 | 92673646 |  | 240 | 108 | 1,18E-12 | 6,20E-11 |
| 2_60557408 | 2 | 60557408 |  | 1080 | 1437 | 1,18E-12 | 6,22E-11 |
| 7_48120490 | 7 | 48120490 | ENSSSCG00000001720 | 234 | 104 | 1,20E-12 | 6,34E-11 |
| 13_33139157 | 13 | 33139157 | ENSSSCG00000011333 | 228 | 100 | 1,22E-12 | 6,41E-11 |
| 6_43734898 | 6 | 43734898 |  | 269 | 128 | 1,24E-12 | 6,50E-11 |
| 1_305486023 | 1 | 305486023 |  | 328 | 170 | 1,30E-12 | 6,81E-11 |
| 5_38418527 | 5 | 38418527 | TMEM19 | 244 | 111 | 1,35E-12 | 7,07E-11 |


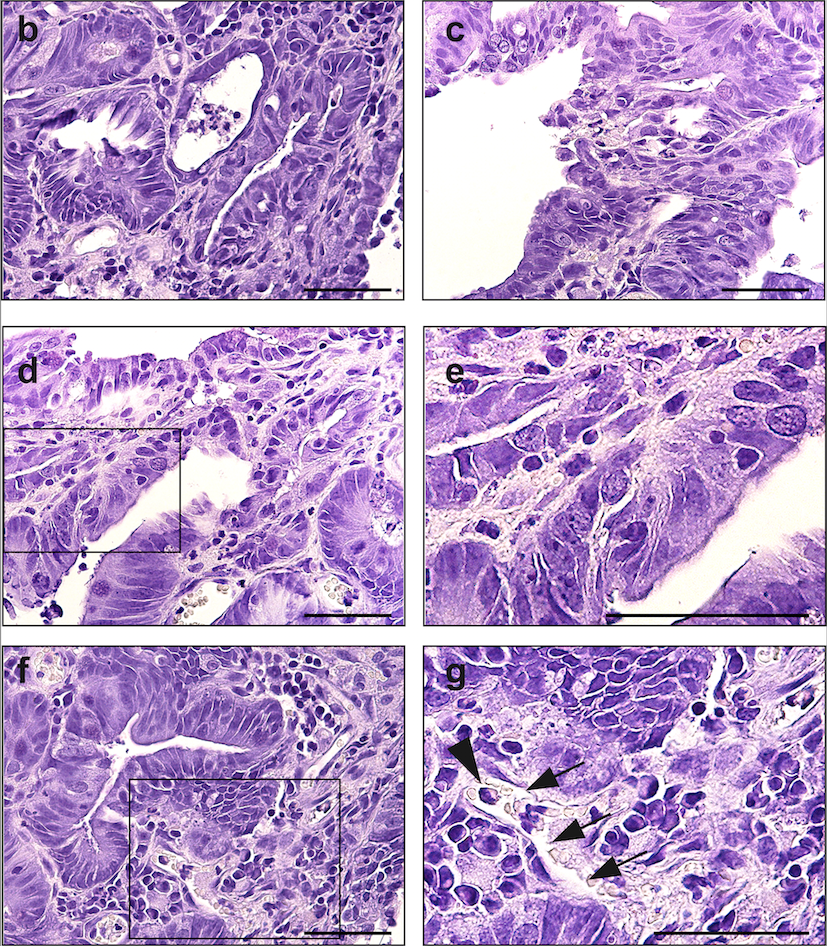


a

b

c

d

e

f

**Supplementary Figure 1.** H&E stained sections of biopsies from the tumour indicated by the arrow in Figure 1a. Arrows in panel (f) indicate a blood vessel with erythrocytes; the arrowhead marks suspected vessel infiltration by a tumour cell. Panels (d) and (f) show indicated high magnification details of panels (c) and (e), respectively. Scale bars, 50µm.


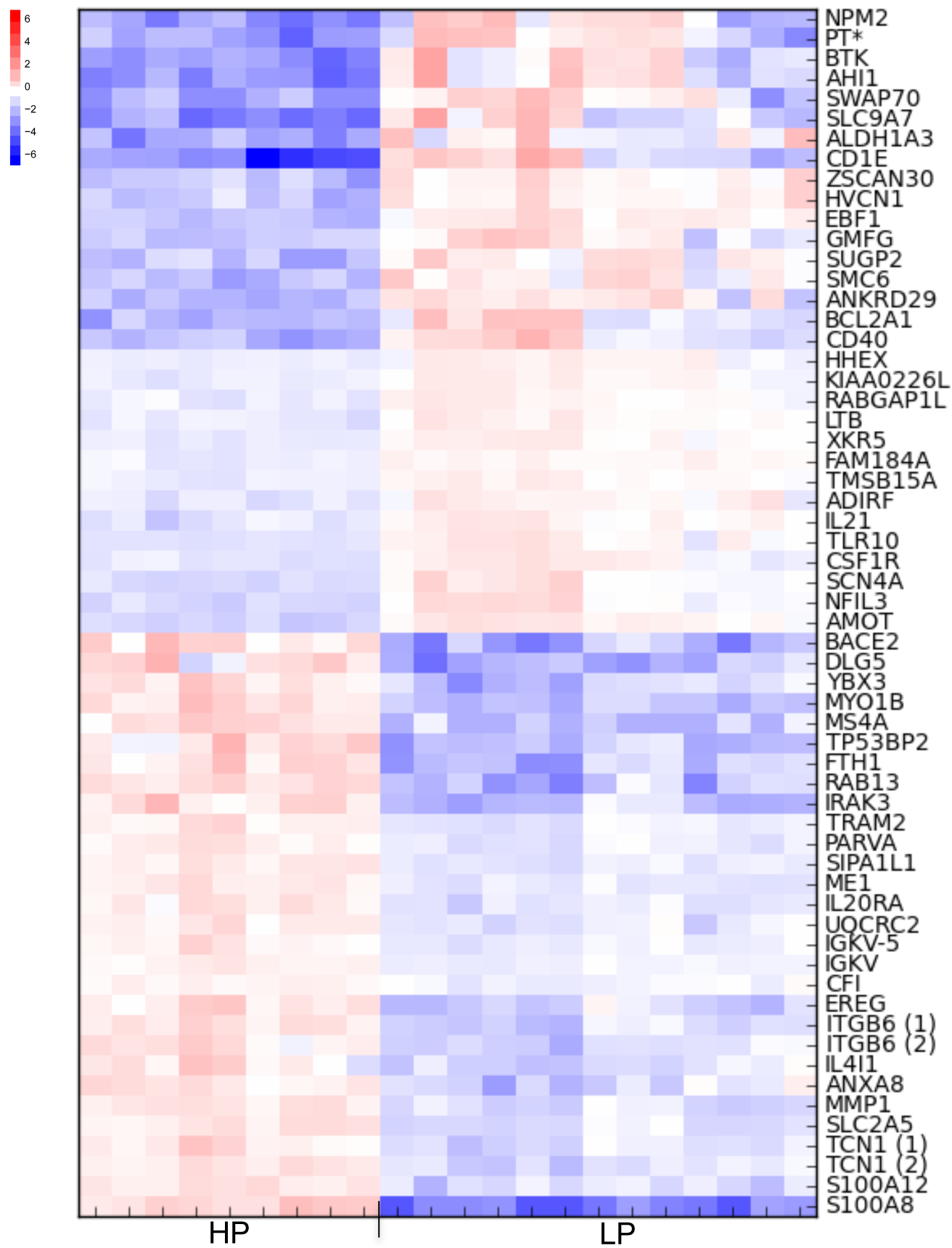


**Supplementary Figure 2**. Heatmap showing unsupervised clustering of histologically unclassified 1 cm diameter polyps taken from 22 *APC^1311/+^* pigs. The top 60 differentially expressed genes correlated with severity of polyposis (LP- low polyp number vs HP- high polyp number) in the source animals.


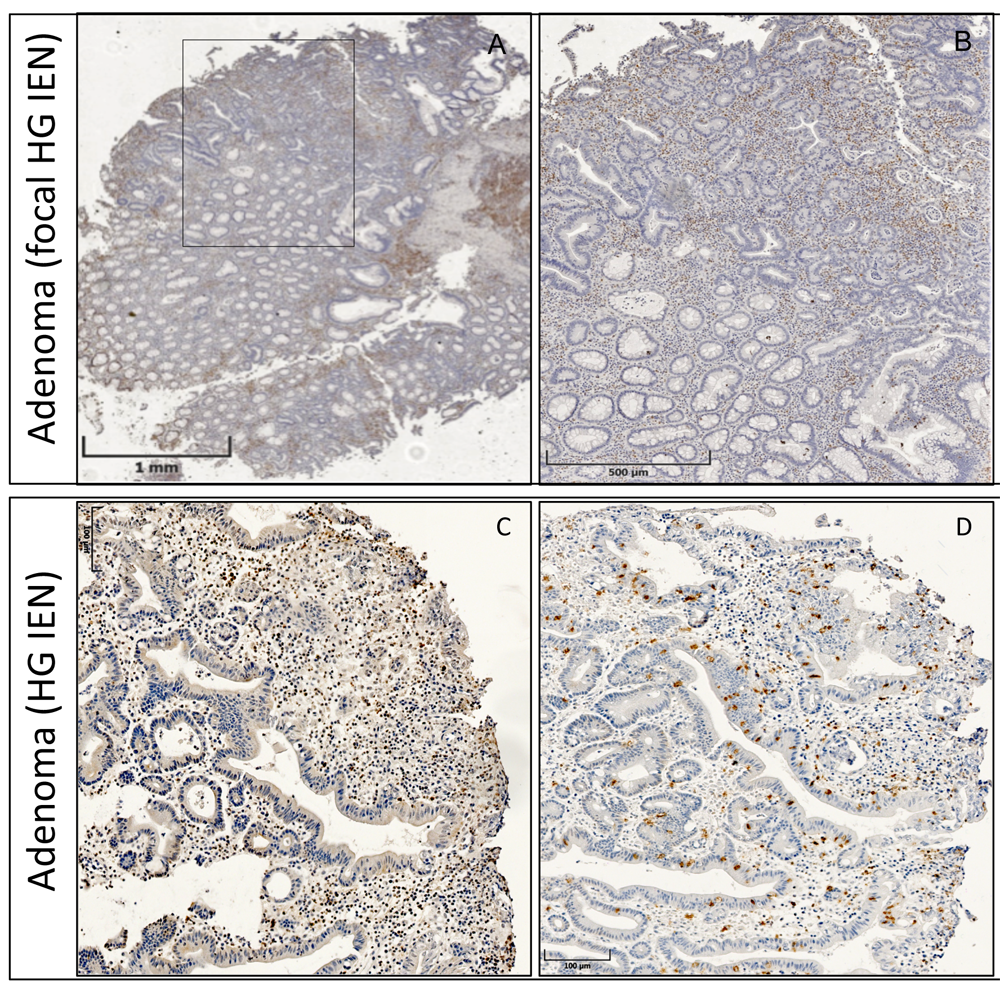


**Supplementary Figure 3.** T cell infiltration of adenomatous polyp. (a) Immunohistochemical staining of CD3^+^ (brown) infiltrating T cells (a, b). Panel (a) shows high magnification details of panel a. Panel c shows CD4^+^ (brown) and panel d CD8^+^ (brown) infiltrating T cells. IEN, high-grade intraepithelial neoplasia.
